# Development and Validation of Open-Source R Package HMCtdm for Therapeutic Drug Monitoring

**DOI:** 10.3390/ph15020127

**Published:** 2022-01-21

**Authors:** Sooyoung Lee, Moonsik Song, Woojae Lim, Eunjung Song, Jongdae Han, Bo-Hyung Kim

**Affiliations:** 1 Department of Life and Nanopharmaceutical Sciences, Graduate School, Kyung Hee University, Seoul 02447, Korea; ls009@khu.ac.kr; 2 Department of Biomedical Science and Technology, Graduate School, Kyung Hee University, Seoul 02447, Korea; sikso1897@gmail.com (M.S.); aidenlim.dev@gmail.com (W.L.); 3 Department of Clinical Pharmacology and Therapeutics, Kyung Hee University Medical Center, Seoul 02447, Korea; yue0327@khu.ac.kr; 4 Department of Computer Science, Sangmyung University, Seoul 03016, Korea; elvenwhite@smu.ac.kr; 5 Department of Biomedical and Pharmaceutical Sciences, Graduate School, Kyung Hee University, Seoul 02447, Korea; 6 East-West Medical Research Institute, Kyung Hee University, Seoul 02447, Korea

**Keywords:** pharmacokinetic, Bayesian method, simulation, MAP, HMC

## Abstract

Most therapeutic drug monitoring (TDM) packages are based on the maximum a posteriori (MAP) estimation. In this study, HMCtdm, a new TDM package, was developed using a Hamiltonian Monte Carlo (HMC) simulation. The estimation process of HMCtdm for the drugs amikacin, vancomycin, theophylline, and phenytoin was based on the R package Torsten. The prior pharmacokinetic (PK) models of the drugs were derived from the Abbottbase^®^ pharmacokinetics systems (PKS) program. The performance of HMCtdm for each drug was assessed through internal and external validations. The internal validation results of the HMCtdm were compared with those of a MAP-based estimation. The developed open-source HMCtdm package is user friendly. The validation results were reviewed and interpreted using the mean percentage error and root mean squared error. The successful transplantation of the prior PK structures (used in PKS) was confirmed by comparing the validation results with a MAP estimation. An open-source HMC-based TDM package was also successfully developed in this study, and its performance was evaluated. This package can be operated by users unfamiliar with C++ and can be further developed for various applications.

## 1. Introduction

An optimal drug dose is crucial for determining therapeutic success. Appropriate drug dosing should be based on the pharmacokinetic (PK) parameters of the individual patient and evaluated from the drug concentrations. Therapeutic drug monitoring (TDM) uses TDM software to estimate PK parameters and predict the drug level according to the specific dosage regimens.

Various programs have been developed for TDM, including InsightRX, PrecisePK, and TDMx, among others. Most of these programs include one- or two-compartment PK models, after intravenous or oral administration, for targeting the therapeutic levels of vancomycin, aminoglycosides, theophylline, or phenytoin [1,2]. TDM software provides PK parameters based on the Bayesian method, where prior information is obtained from PK data of the previous reference study. Thus, despite the limited clinical data obtained from individual patients, these informative priors can enable the estimation of PK parameters [3,4,5].

The most frequently used Bayesian methodology for TDM is the maximum a posteriori (MAP) procedure, which estimates the posterior mode using the data of an individual patient and prior distributions [6]. Although the MAP estimation method was developed 50 years ago, it is still dominantly used in the estimation of PK parameters. Since computation is relatively straightforward, the MAP is still a useful method for estimating the posterior mode.

However, more information on the posterior distribution, including the posterior mode, can be obtained using a full Bayesian analysis. A full Bayesian analysis is conducted using methods, such as a Markov chain Monte Carlo (MCMC) approach, which require numerous computations. Thus, for ease of calculation, Bayesian MCMC algorithm software–such as WinBUGS and Jags based on Gibbs sampling and Stan based on a Hamiltonian Monte Carlo (HMC) simulation—has been developed constantly [7,8,9].

The development of new algorithms and an improvement in the computational speed has led to a full Bayesian analysis of the PK data [10]. Studies on PK analysis using a full Bayesian estimation have been reported [11,12,13,14]. However, most studies have focused on estimating the individual parameters after conducting a population PK analysis rather than on a TDM study in which individual PK parameters are estimated using a Bayesian analysis without an estimation of the population PK parameter [11,12]. Furthermore, to the best of our knowledge, no TDM package has been developed to date using an HMC-based MCMC algorithm.

**Figure 1:**
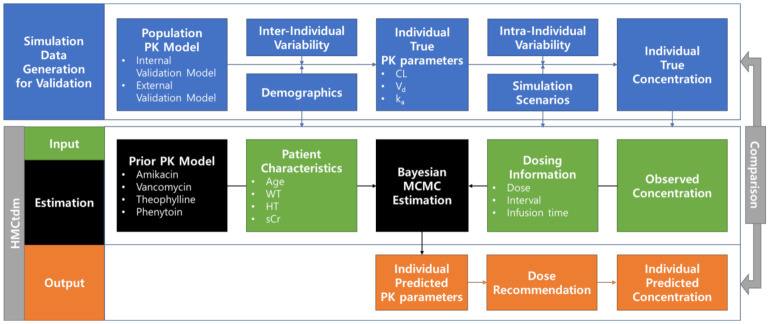
Overview of the current study. The process of generating simulation data for validation is shown in the blue box. The three parts (input, estimation, and output) of the HMCtdm workflow are distinguished by the green, black, and orange boxes, respectively. The true values generated are compared for validation with the values predicted by HMCtdm. Abbreviations: CL, clearance; V_d_, volume of distribution; ka, first-order absorption rate constant; WT, body weight; HT, height; sCr, serum creatinine.

The current study was conducted to develop HMCtdm, a new MCMC-based TDM package, by transplanting the population PK model of an existing MAP-based package, the Abbottbase^®^ PK system. In addition, the package was validated using four different drugs and blood samples withdrawn at various time points. The overview of the current study is presented in Figure 1.

## 2. Results

The developed package can be divided into three parts: input, estimation, and output (Figure 1). In the input, the package requires the entry of a table containing the patient characteristics, dosing information, and observed drug concentration. The format of the table is similar to that of the NONMEM software dataset. The variables that require default inputs are as follows: individual identification number (*ID*), the event times of dosing or concentration observation (*time*), event indicator (*evid*) (e.g., 0 = observation, 1 = dosing), dose administered for dosing event (*amt*), compartment of dosing or concentration observation (*cmt*), rate of infusion (*rate*), dosing interval (*ii*), and number of additional doses (*addl*). The following R code provides example input data for amikacin, i.e., *get_sample_data(drug=“amikacin”)*.

In the estimation, PK parameters are estimated from input data and prior models using Torsten based on MCMC [15]. For example, in case a user wants to estimate the PK parameter for amikacin from the input data, *data_set*, the following code is input: *hmctdmrest(drug=“amikacin”, data=“data_set”)*. The prior PK models of the four drugs for estimation were included in the package. Default prior information can be checked in the package. For example, to view the prior models of amikacin, the following code is input: *get_default_prior(drug=“amikacin”)*.

In the output, the estimated PK parameter, concentration at the desired time, and recommended dose are presented. The concentration at the desired time was calculated from the estimated PK parameter using mrgsolve [16]. The recommended dose is calculated by the following code: *get_recommended_dose(mode=mode, target=target, current_dose=current_dose, current_status=current_status, …)*, where *mode* is the type of target value (e.g., $C_{t,\;target}^{ss}$, $AUC_{\tau ,\;target}^{ss}$, ${\bar{C}}_{target}$), *target* is target value, *current_dose* is the dose amount in the current dosage regimen, and *current_status* is the target value when the current regimen is maintained. HMCtdm is provided in a repository at https://github.com/SikSo1897/hmctdm/tree/develop (accessed on 1 November 2021). A detailed description of the input data preparation, estimation, and output production are described in the README of the repository.

**Figure 2:**
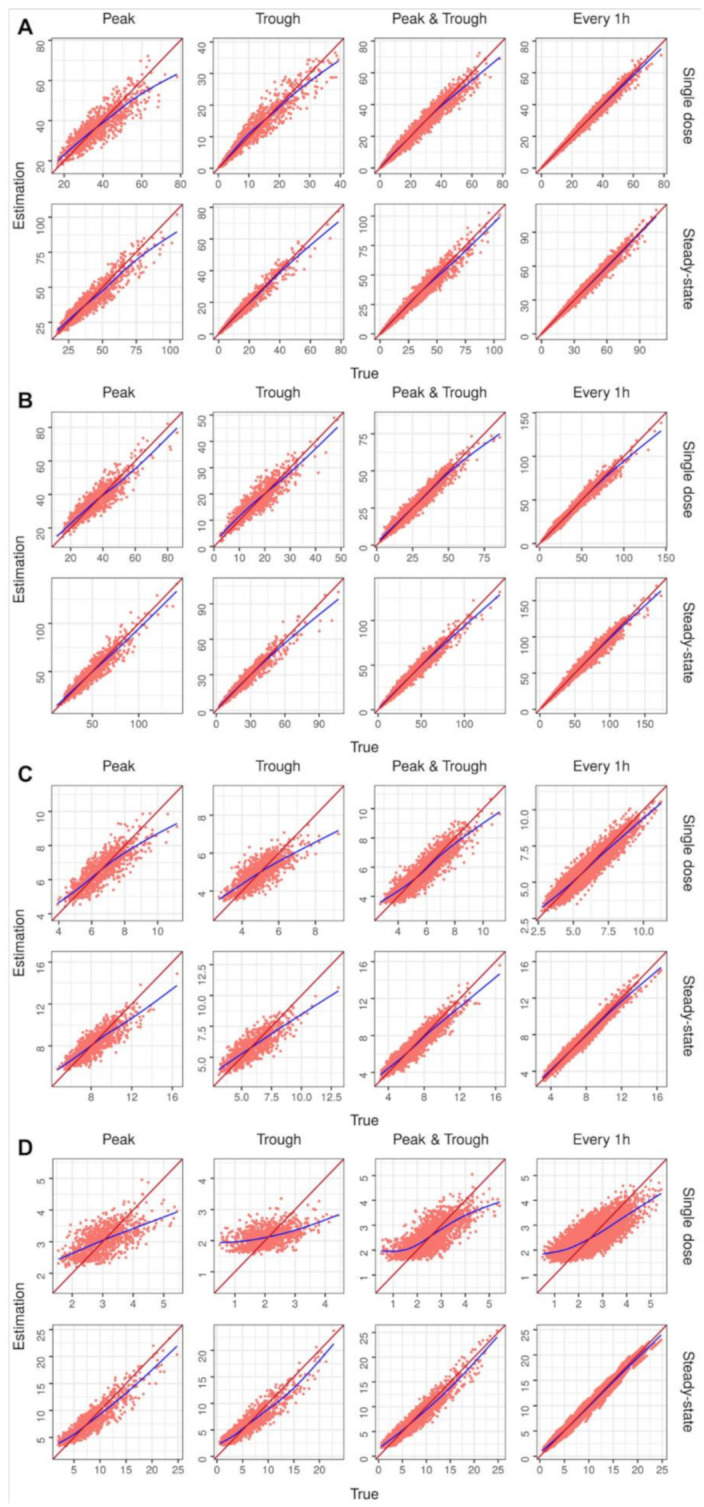
Graphs of estimated versus true concentration for each internal validation scenario. The identity line is shown in red, and a trend line in blue has been drawn for each model: (**A**) amikacin, (**B**) vancomycin, (**C**) theophylline, and (**D**) phenytoin.

The internal validation for package performance was conducted using a total of 32 scenarios for four drugs, four sampling point sets, and two dose cases. A total of 32,000 virtual patients were tested, i.e., 1000 for each scenario. Table 1 shows the estimation performance calculated using MPE and RMSE for the concentration of drugs under several scenarios. Figure 2 shows a plot of the true versus estimated values of the drug concentration. The results of the estimated individual PK parameters for each drug are shown in Tables S1–S4 and Figures S1–S4.

**Figure 3:**
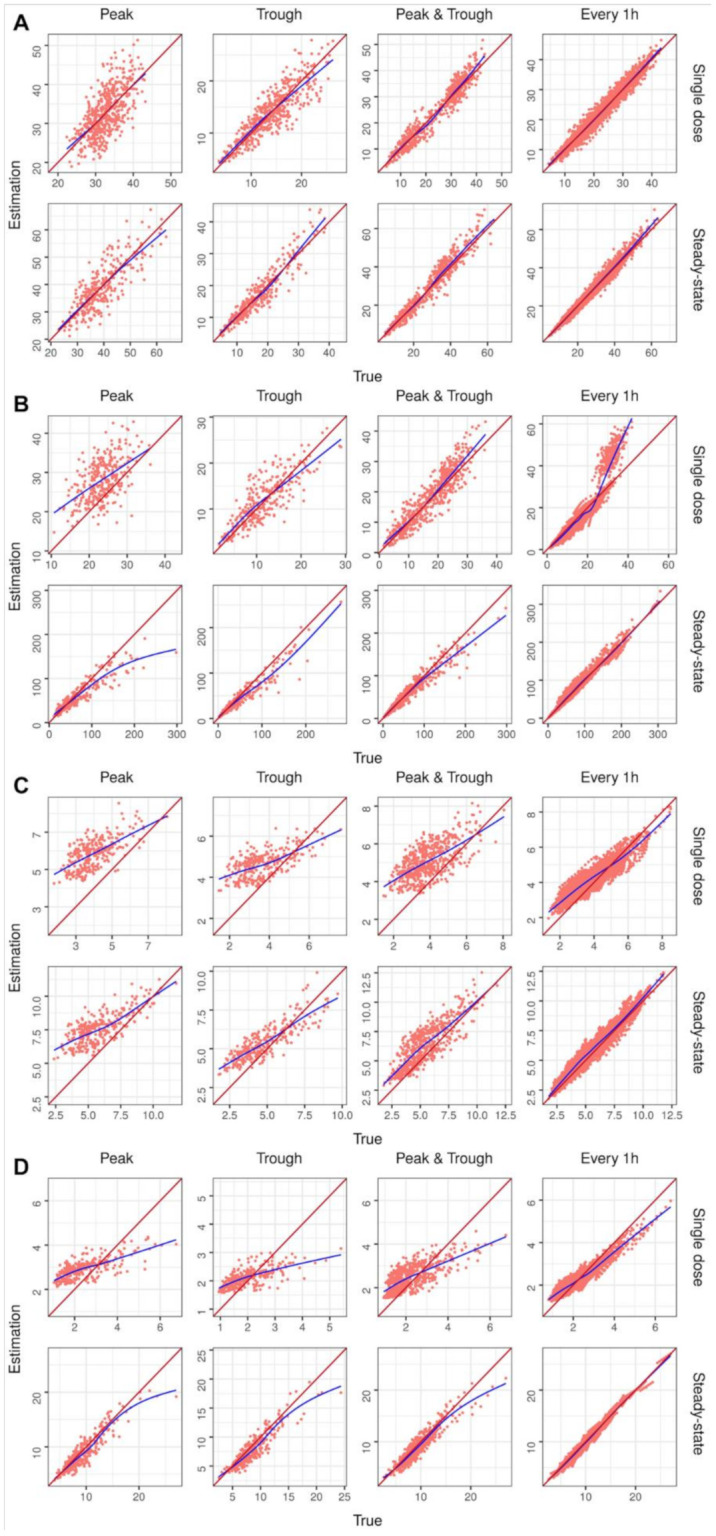
Graphs of estimated versus true concentration for each external validation scenario. The identity line is shown in red, and a trend line in blue has been drawn for each model: (**A**) amikacin, (**B**) vancomycin, (**C**) theophylline, and (**D**) phenytoin.

The external validation was conducted on a total of 9600 virtual patients, i.e., 300 each for the same scenarios applied in the internal validation. Table 2 shows the performance of the concentration estimation of the drugs for each scenario used in the external validation. Figure 3 shows the true concentration versus the estimated concentration in the external validation. The external validation results based on the parameter estimation for each drug are shown in Tables S5–S8 and Figures S5–S8.

**Figure 4:**
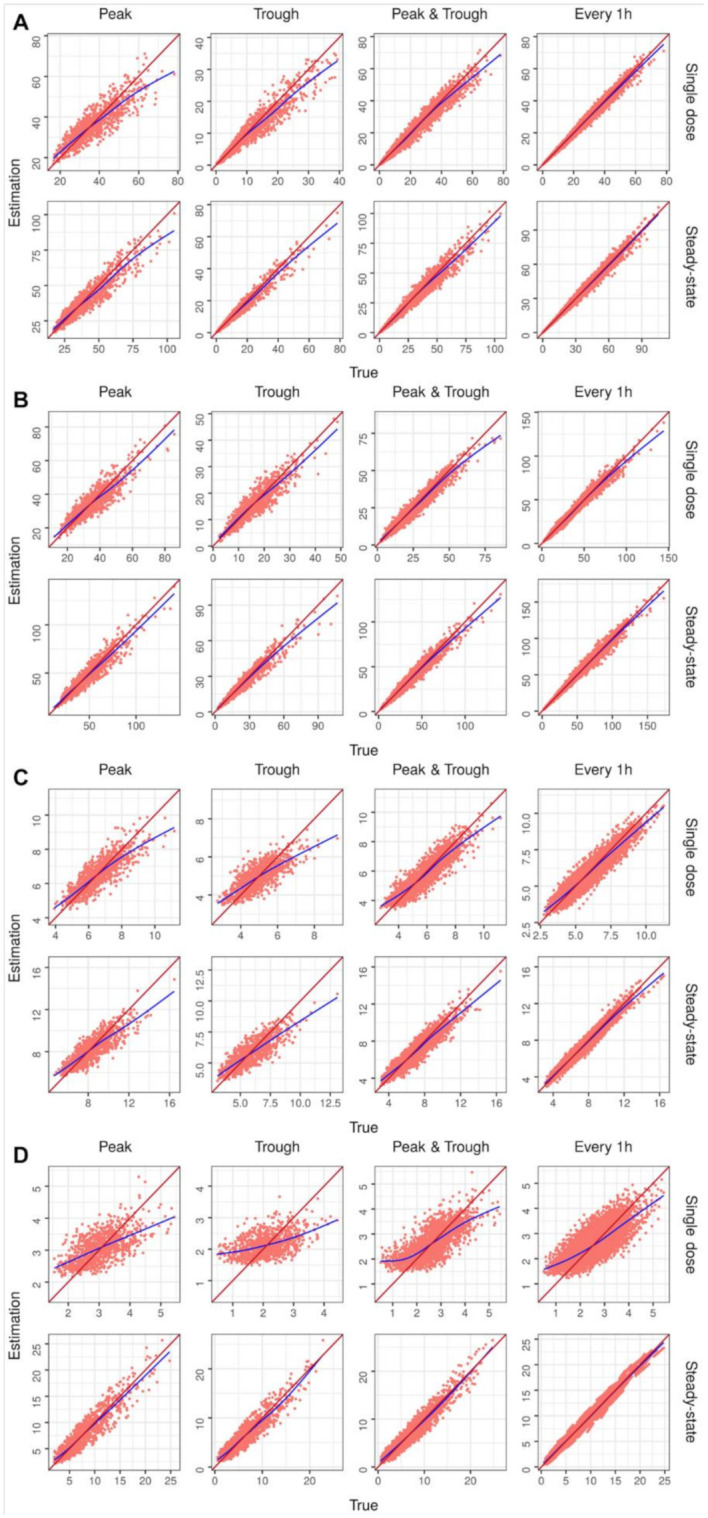
Graphs of estimated versus true concentration for each internal validation scenario using a MAP estimation. The identity line is shown in red, and a trend line in blue has been drawn for each model: (**A**) amikacin, (**B**) vancomycin, (**C**) theophylline, and (**D**) phenytoin.

A MAP estimation was applied to a total of 32,000 virtual patients using the same scenario and data as in the internal validation. Table 3 and Figure 4 show the results of the concentration estimation for the different drugs under each scenario in which the MAP estimation was conducted. The estimated results of the individual parameters using the MAP estimation are shown in Tables S9–S12 and Figures S9–S12.

## 3. Discussion

HMCtdm was developed as an open-source R package for TDM with pharmacokinetic models. This helps users unfamiliar with C++ and Stan programs to apply the TDM workflows (which utilize Stan as a simple input) and, thereby, to estimate the parameters and calculate the drug concentration at the desired time. As the source code of the estimated model utilizes the ODE System of the Stan and Torsten library, there is no need to transform the ODE into a complex closed form. HMCtdm also contains validated PK models from the PKS. Among the various PKS drugs, PK models with different characteristics, such as one or two compartments, were included in the package. Although the package does not support the creation of a new PK model, its workflow is simple, and the model can be modified into a base for creating a new model. As needed, the PK models of the source code can be applied to new simulation studies. Since HMCtdm is based on Stan using C++ syntax, it can be extended to other programming languages and be developed into various types of TDM software as it can be combined with programming languages, such as Python, shell, MATLAB, and R.

Internal validation was conducted to test the performance of the HMCtdm package. For amikacin, the prediction of the concentrations under all scenarios appeared to be good in terms of bias (as MPE) and precision (as RMSE). Although the prediction of the concentration was good (Table 1, Figure 2), the estimation of the individual parameters was poor (Table S1, Figure S1). In the single-dose case, the estimate of V_nr_ was better in the peak sample set than in the trough sample set. In contrast, the estimate of CL_slope_ was better in the trough sample set than in the peak sample set. This was due to the differences in the information of the parameters at each time point of the concentration [17]. As the number of sampling points increased, the estimation results of CL_slope_ and V_nr_ improved, whereas those of CL_nr_ did not. In 1000 simulations of patients, the true mean of the product of CL_nr_ and LBW was 2.5 mL/min, and the product of CL_slope_ and CrCl was 45.3 mL/min. Therefore, the influence of CL_nr_ on the clearance (CL) is not substantial and can be estimated regardless of the simulation scenarios. Estimated CL_slope_ showed better results at steady state than after a single dose, because the concentration under a steady state is influenced more by the CL than the volume of distribution (V) [18]. Thus, on reaching steady state, the concentration gave more information regarding the CL than the V.

Vancomycin showed a poor overall estimation performance for the single-dose cases (Table 1 and Table S2, Figure 2 and Figure S2). For the individual parameters (Table S2, Figure S2), the range of the estimated value did not change as much as the true parameter. Since the number of parameters to be estimated increases for vancomycin when compared with that for amikacin, the information based on the concentration would be weaker than prior information. The estimated CL_slope_ showed better results at steady state than after a single dose, as observed in the case of amikacin. Therefore, in comparison to single-dose cases, an improvement in the concentration prediction performance was observed.

Theophylline was assumed to be a sustained-release drug, and *k_a_* was set to 0.27 h^−1^, and thus, the estimated PK parameters were generally poor (Table S3, Figure S3). Based on the prior PK model, the time of peak concentration was at 6.40 h with CL_nr_ at 40 mg/h/kg and V_nr_ at 0.4 L/h. As the peak sampling time was set to a 4 h in the validation, blood samples at the true peak point were not collected, and the value of V could not be estimated. In addition, flip–flop kinetics can be assumed in the sustained-release formulation. Because the elimination rate depends on k_a_, it would be difficult to calculate using CL/V. Therefore, although the number of input concentrations increased, the estimate could not be calculated. The estimation of CL_nr_ was improved under a steady state when compared with a single case. It is assumed that as the number of input concentrations increases the correct CL_nr_ is estimated, thus improving the estimation of V_nr_.

For the single-dose cases of phenytoin, the overall estimation performance was poor in terms of the both the concentration and PK parameters (Table 1 and Table S4, Figure 2 and Figure S4). Although the number of input concentrations increased, the estimations of V_max_ and k_m_ showed little change, and V_nr_ showed a slight improvement. The estimation of the concentration and V_max_ were substantially improved at steady state compared to that after a single dose, and the performances of k_m_ and V_nr_ were biased. The PK model of phenytoin assumes Michaelis–Menten kinetics. Therefore, owing to the relatively low concentration in the single-dose cases (when compared with a steady state), both V_max_ and k_m_ were involved in determining CL, necessitating the information split for the estimation of both parameters related to CL (again, when compared with a steady state). Under a steady state, CL is determined by V_max_, which can lead to an estimation of V_max_ with more information related to CL (when compared with a single-dose case). Thus, under a steady state, the estimation performance of V_max_ was substantially improved, and the prediction of the concentration was thus improved. Nevertheless, the estimation results of phenytoin showed bias compared with that of other drugs, particularly in the single-dose cases. This could be an effect of the component values of the intra-individual variability, determined through the following equation: $\sigma =CV_{assay}\cdot C_{Pred}+S_{assay}$. In our study, $S_{assay}$ was 1.0 mg/L for phenytoin, which was higher than 0.25 mg/L for the other drugs (Table 4). As a result, the observed concentrations of phenytoin could have a relatively high intra-individual variability, particularly at low concentrations. The observed concentrations used in the estimation can deviate from the true values owing to the high intra-individual variability. Consequently, the estimation performance may have poor results from the high $S_{assay}$, particularly under low concentrations of single-dose phenytoin cases.

As the number of blood sampling points increased, the estimation performance generally improved. However, since the estimation of all parameters was not improved by increasing the number of blood sampling points, it is necessary for estimation to use the proper number and time points of blood samples specified by each PK model of a drug. Thus, an appropriate TDM strategy can be devised by referring to the validation results of this study. For example, to determine the initial dose of vancomycin, blood can be collected immediately at the peak point after the first administration, and the loading dose from the second administration can be corrected. This is because sampling at the peak point shows a better estimate of V than that at the trough. Thereafter, it can be considered to determine the maintenance dose through blood sampling at the trough, which gives a better estimation of CL.

After external validation, it was observed that the estimation slowly declined in performance when compared with that of the internal validation (Table 2 and Tables S5–S8, Figure 2 and Figures S5–S8). Since TDM uses a Bayesian estimation, the estimation result is affected by the prior information. Therefore, the estimation of the external validation using a PK model with a different population is more inaccurate than the internal validation using the same model. In conclusion, it is important to select a suitable prior for a better predictability of the individual PK parameter. Various studies about priors have been reported to improve the TDM performance, including a study on finding a population PK model with a better predictive performance, study applying a non-informative prior for TDM, and study by attempting to model the selection/averaging [3,4,5]. If the results of these studies are accumulated, a better prior can be applied to HMCtdm.

The overall MAP estimation results were similar to those of the MCMC estimation (Table 3 and Tables S9–S12, Figure 3 and Figures S9–S12). All PK parameters were generated and estimated from log-transformed normal distributions. Therefore, it was assumed that the MAP (which calculates the posterior mode) and MCMC (which calculates the posterior median) produced similar estimates [6,15]. Most of the estimation algorithms of the TDM package are based on a MAP, whereas HMCtdm is based on the MCMC algorithms [19]. Unlike MAP, MCMC can estimate the variance [13,14]. In particular, HMC is considered as the gold standard among many Monte Carlo sampling methods [12]. Therefore, further research on the application of variance, estimated using MCMC, for clinical purposes will be needed in the future.

Although this study focused on the development of a new TDM package and various validations, it has certain limitations. The package can calculate the dose target values, such as $C_{t,\;Dose}^{ss}$, $AUC_{\tau ,\;Dose}^{ss}$, and ${\bar{C}}_{Dose}$, but does not suggest calculating the specific target value corresponding to each drug. It allows calculating several target values through a single estimation. For example, according to need, both the predicted target area under the curve (*AUC*) of the time–concentration curve and trough concentration can be calculated for vancomycin. Although this can increase the autonomy, the users who want to promptly know a specific target value of a drug may find this inconvenient. In the validation, first, interpretation of the effects of the simulation parameters, such as the inter- and intra-individual variability, for the PK parameter and concentration in the results is limited. The results in our study showed that the estimation improves as the number of samples increases. Through an external validation, it was observed that the estimation results worsen with the different PK models between generation and estimation. However, there is a limitation in interpreting which simulation parameter has a greater effect on the estimation in this study. Second, phenytoin with nonlinear kinetics could not be validated at various doses. Because the response of nonlinear kinetics drugs is sensitive to changes in drug dose, validation of various doses is required. However, as the simulation scenario was structured when considering the quantity of the information under the different sampling times of each drug, a scenario with a change in dosing regimen could not be included. Finally, all validations were based on simulations. To generate data close to that of an actual patient, the demographics were generated using internal data, and the dosing scenario was set by reviewing the drug approvals and dosing guidelines for each drug. However, validation was not performed using plasma concentration data obtained from actual patients. Therefore, further studies overcoming these limitations can help the package improve and the individual PK parameters to achieve a better estimation.

## 4. Materials and Methods

### 4.1. Development

#### 4.1.1. Package Development

The HMCtdm package was developed based on the R language and runs in R (version 4.1.0, Vienna, Austria) [20]. Based on the number of compartments, administration route, and elimination kinetics, the drugs amikacin, vancomycin, theophylline, and phenytoin were selected in the estimation package. The estimation of individual PK parameters is based on the Bayesian method. The population PK parameter models for the priors of the Bayesian estimation were obtained from the existing commercial program used in PKS. Individual PK parameters are estimated using an HMC simulation, which is an algorithm using in MCMC simulations [21,22].

#### 4.1.2. Pharmacokinetic Model

Table 4 shows the details of the PK parameters of each drug obtained from Abbottbase^®^ PKS (version 1.10, Abbott Laboratories, PKS, Chicago, IL, USA). Intravenous infusion with one- and two-compartment elimination models were applied to both amikacin and vancomycin, and a one-compartment oral administration model was applied to both theophylline and phenytoin. However, theophylline and phenytoin exhibited first-order elimination with first-order absorption and nonlinear elimination with a zero-order absorption, respectively.

The PK parameters were assumed to follow a log normal distribution. Interindividual variability of the PK parameters was converted from the coefficient of variance into the standard deviation. The concentrations were assumed to follow normal distribution. The intra-individual variability of the concentration error model reflected the assay coefficients of variation and the assay sensitivity. The lean body weight (LBW), which is a covariate of several PK parameters, was estimated using Peck’s formula [23]. The creatinine clearance (CrCl) was calculated using the Cockcroft–Gault LBW [24].

#### 4.1.3. Estimation Method

The PK parameters were estimated using an HMC simulation. Model estimation based on the HMC algorithm was conducted using Torsten (version 0.89.0; Metrum Research Group LCC, Tariffville, CT, USA), which is a Stan-based R package that uses an ordinary differential equation (ODE) to estimate the PK parameter [15]. For estimating the PK parameter, four chains were initialized and run for 5000 iterations each (2500 for warmup and 2500 as samples from the posterior). The posterior median of the individual parameters was used as an estimate.

#### 4.1.4. Dose Target and Recommendation

The dose target was computed for $C_{t,\;Dose}^{ss}$, $AUC_{\tau ,\;Dose}^{ss}$, and ${\bar{C}}_{Dose}$. The target $C_{t,\;Dose}^{ss}$ is the steady-state concentration at time t after administration of the amount of Dose. The target $AUC_{\tau ,\;Dose}^{ss}$ is the *AUC* of the time–concentration during the dosing interval τ when the amount of Dose is administered under a steady state. The target ${\bar{C}}_{Dose}$ is the average concentration under a steady state when the amount of Dose is administered. In addition, $C_{t,\;Dose}^{ss}$ and $AUC_{\tau ,\;Dose}^{ss}$ were predicted from the individual estimated PK parameters using mrgsolve [16], and ${\bar{C}}_{Dose}$ was calculated as follows:

$${\bar{C}}_{Dose}=\frac{AUC_{\tau ,\;Dose}^{ss}}{\tau }$$

where $AUC_{\tau ,\;Dose}^{ss}$ is the dose target *AUC* calculated using mrgsolve, and τ is the dosing interval.

The dose recommendation was computed for a dose that can achieve the therapeutic target of the drug under steady state. When dose target is under steady-state concentration at time t, which is $C_{t,\;target}^{ss}$, the recommended dose is calculated as follows:

$$Recommended\;Dose=\frac{Current\;Dose}{C_{t,\;current\;dose}^{ss}}\times C_{t,\;target}^{ss}$$

where Current Dose is the currently administered dose, $C_{t,\;current\;dose}^{ss}$ is the predicted steady-state concentration at time t after administration when the Current Dose is maintained, and $C_{t,\;target}^{ss}$ is the target concentration at time t under steady state. In addition, $C_{t,\;current\;dose}^{ss}$ is calculated using mrgsolve, and $C_{t,\;target}^{ss}$ is specified by the user. When the dose target is the *AUC* during τ under steady state, i.e., $AUC_{\tau ,\;target}^{ss}$, the recommended dose is calculated as follows:

$$Recommended\;\mathrm{Dose}=\frac{Current\;Dose}{AUC_{\tau ,\;current\;dose}^{ss}}\times AUC_{\tau ,\;target}^{ss}$$

where $AUC_{\tau ,\;current\;dose}^{ss}$ is the predicted *AUC* during τ under steady state when the Current Dose is maintained, and $AUC_{\tau ,\;target}^{ss}$ is the target *AUC* during τ under steady state. In addition, $AUC_{\tau ,\;current\;dose}^{ss}$ is calculated by mrgsolve, and $AUC_{\tau ,\;target}^{ss}$ is specified by the user. When the dose target is the average concentration during τ under steady state, which is ${\bar{C}}_{target}$, the recommended dose is calculated as follows:

$$Recommended\;\mathrm{Dose}=\frac{Current\;Dose}{{\bar{C}}_{current\;dose}}\times {\bar{C}}_{target},$$

where ${\bar{C}}_{current\;dose}$ is the predicted average concentration during τ under steady state when the Current Dose is maintained, and ${\bar{C}}_{target}$ is the average target concentration during τ under steady state. In addition, ${\bar{C}}_{current\;dose}$ is calculated by dividing $AUC_{\tau ,\;current\;dose}^{ss}$, computed using mrgsolve, by τ, and ${\bar{C}}_{target}$ is specified by the user.

### 4.2. Validation

The estimation performance was validated through simulation tests. The process of generating the simulation data for validation was based on a population PK model (Figure 1). Individual true PK parameters were generated by integrating the interindividual variability and demographic characteristics in the population PK model. The true concentrations were calculated from the individual PK parameters under each simulation scenario. The observed concentrations were generated by incorporating the intra-individual variability into the true concentrations. The R package mrgsolve (version 0.11.2; Metrum Research Group LCC, Tariffville, CT, USA) was used to generate the simulation data for validation [16]. The PK models differed for the internal and external validation.

Internal data from the Kyung Hee University Hospital Clinical Trial Center were used to generate the patient demographics, and the mean ± SD for height (cm), weight (kg), and age (years) were calculated as 165.1 ± 8.7, 65.1 ± 10.2, and 50.2 ± 17.1, respectively. Table 5 shows a simulation scenario of the drug dosage regimen and the blood sampling time points for the test drugs. The dosage regimen was based on the drug label provided by the Ministry of Food and Drug Safety in Korea (MFDS) [25,26,27,28]. The blood sampling time points were referenced from the TDM guidelines of each drug [29,30,31]. To avoid complexities in the validation process, the dosage regimen and timings of the blood samples were slightly modified. To examine the various estimations, the blood sampling point was set to four cases: peak, trough, peak and trough, and 1 h intervals, which were applied for both single-dose and steady-state timings.

For validating the HMCtdm estimation, the simulation data of the demographic, dosing scenario, and observed concentration were used as the input. The value estimated by HMCtdm was compared with the true value.

#### 4.2.1. Internal Validation PK model

The PK model of the simulated patient for internal validation was generated from the same structure as the PKS model used for estimation (Table 4).

#### 4.2.2. External Validation of PK model

The PK model of the simulated patients for the external validation was based on a reported population pharmacokinetic study for each drug (Table 6) [32,33,34,35]. Articles on population pharmacokinetic studies of Korean patients were selected for amikacin and vancomycin, whereas in the absence of appropriate Korean adult subject studies, Japanese articles were selected for studying theophylline and phenytoin. To simplify the model, k_a_ of theophylline was fixed. It was assumed that none of the patients suffered from any underlying disease and the use of any concomitant drugs was absent. The external validation of the PK models is shown in Table 6.

#### 4.2.3. Performance Evaluation

The validations of the estimations were assessed based on the mean percent error (MPE) and root mean squared error (RMSE) of the prediction values of each simulation set relative to the observed values, which are defined as follows:

$$MPE=\frac{1}{N}\sum_{i=1}^{N}\frac{EST_{i}-TRUE_{i}}{TRUE_{i}}\times 100\%,$$

$$RMSE=\sqrt{\frac{1}{N}\sum_{i=1}^{N}{\left(EST_{i}-TRUE_{i}\right)}^{2}},$$

where $EST_{i}$ is the estimated value, $TRUE_{i}$ is the corresponding true value for individual *i*, and *N* is the number of patients. The values quantitatively express the PK parameters and the drug concentrations.

**Figure 5:**
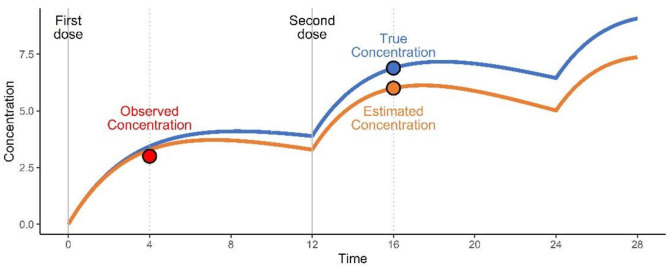
An illustrative example of evaluated concentration of theophylline. The blue and orange lines are the time–concentration profiles of the true and estimated values, respectively. The red dot represents the observed concentration collected at peak time after the first dose. The blue and orange dots represent the true and estimated concentrations calculated at the peak time after the second dose, respectively.

The estimated concentrations were calculated using estimated individual PK parameters and using the time points after one dosing interval from the observed concentration (Figure 5). The true concentration was calculated using the true PK parameters and using the time points equal to the estimated concentration. The prediction error was not reflected in the true concentration value for comparison. The steady-state concentrations were calculated using the *ss* option of mrgsolve for all drugs except phenytoin. The steady state of phenytoin was assumed at the 20th dose as this level could not be reached using the *ss* option of mrgsolve in many cases. Therefore, for phenytoin at steady-state, the observed concentration at the 20th dose and the true and estimated concentrations at the 21st dose were calculated.

For the internal validation, the MPE and RMSE of each PK parameter were calculated directly because the generated and estimated models corresponded. The external validation of the PK model parameters differed from that of the estimated PK model. Therefore, the individual clearance, volume of distribution, maximum velocity, and Michaelis constant were recalculated for calculating the MPE and RMSE during the external validation.

### 4.3. MAP Estimation

A MAP estimation was conducted (under the same scenario as the internal validation) to verify whether the developed package provided an appropriate estimation of the PK parameters. The MAP objective function is defined as

$$\Phi =\sum_{i=1}^{i=N}\frac{{\left(C_{OBS_{i}}-C_{EST_{i}}\right)}^{2}}{{\hat{\sigma }}_{i}{}^{2}}+\sum_{k=1}^{k=L}\frac{{\left(\ln P_{mean_{k}}-\ln P_{EST_{k}}\right)}^{2}\;}{{\hat{\omega }}_{k}{}^{2}},$$

where $C_{OBS_{i}}$ is the observed concentration, $C_{EST_{i}}$ is the predicted concentration, and σ is the intra-individual variability of the concentration for the *i*-th concentration of a total of *N* measured concentrations [6]. In addition, $P_{mean_{k}}$ is the mean of population PK parameter, $P_{EST_{k}}$ is the estimated individual PK parameter, and ω is the interindividual variability of the PK parameters for the *k*-th parameter of a total of *L* parameters. The MAP estimation was conducted using the R package mapbayr [36].

## 5. Conclusions

In this study, a new HMC-based TDM package was developed, and its performance was evaluated under various simulation scenarios. The validation results were carefully reviewed, and the package confirmed the successful transplantation of the prior PK structures using PKS. This open-source package was developed for users unfamiliar with the C++ programming language and can be further developed and applied for various purposes in the future.

## Figures and Tables

**Table 1:** Performance of internal validation data estimation of concentration prediction.

Sampling Time	Peak		Trough		Peak and Trough		Every 1 h	
	MPE (%)	RMSE (mg/L)	MPE (%)	RMSE (mg/L)	MPE(%)	RMSE (mg/L)	MPE(%)	RMSE (mg/L)
Amikacin								
Single dose	0.91	5.11	4.92	2.48	1.37	3.22	−0.32	1.35
Steady state	−0.85	5.31	2.14	2.49	0.70	3.81	−0.35	1.75
Vancomycin								
Single dose	2.32	4.51	6.72	2.85	2.27	3.06	0.23	2.04
Steady state	0.39	6.12	1.89	3.80	1.12	4.14	−0.11	2.49
Theophylline								
Single dose	−0.01	0.61	1.56	0.59	0.78	0.58	−0.03	0.40
Steady state	−0.25	0.85	0.90	0.77	−0.18	0.68	−0.45	0.35
Phenytoin								
Single dose	3.54	0.52	12.04	0.56	7.53	0.53	5.28	0.46
Steady state	5.28	1.60	13.91	1.52	7.24	1.18	2.27	0.58

**Table 2:** Performance of external validation data estimation of concentration prediction.

Sampling Time	Peak		Trough		Peak and Trough		Every 1 h	
	MPE (%)	RMSE (mg/L)	MPE (%)	RMSE (mg/L)	MPE(%)	RMSE (mg/L)	MPE(%)	RMSE (mg/L)
Amikacin								
Single dose	0.25	4.44	1.15	2.34	0.75	2.86	−0.07	1.62
Steady state	−0.14	5.14	−0.10	2.72	1.86	3.49	0.32	1.83
Vancomycin								
Single dose	21.91	6.66	6.99	2.95	5.49	3.45	−5.25	4.47
Steady state	−2.72	21.19	−5.08	15.92	−0.62	13.11	3.38	6.60
Theophylline								
Single dose	53.35	2.04	34.62	1.29	37.92	1.48	12.53	0.60
Steady state	37.15	2.09	19.43	1.07	21.26	1.37	6.93	0.58
Phenytoin								
Single dose	34.39	0.91	14.65	0.57	21.15	0.70	8.07	0.34
Steady state	−5.13	1.43	−8.01	1.44	−4.36	1.16	−1.96	0.54

**Table 3:** Performance of MAP estimation of concentration prediction.

Sampling Time	Peak		Trough		Peak and Trough		Every 1 h	
	MPE (%)	RMSE (mg/L)	MPE (%)	RMSE (mg/L)	MPE(%)	RMSE (mg/L)	MPE(%)	RMSE (mg/L)
Amikacin								
Single dose	−0.33	5.16	−4.65	2.72	−2.28	3.34	−1.16	1.38
Steady state	−1.85	5.41	−2.64	2.66	−1.93	3.89	−0.84	1.77
Vancomycin								
Single dose	−0.15	4.53	0.22	2.82	−1.20	3.14	−1.16	2.06
Steady state	−0.97	6.17	−1.79	3.94	−1.26	4.22	−0.80	2.46
Theophylline								
Single dose	−0.26	0.61	1.22	0.59	0.48	0.58	−0.31	0.40
Steady state	−0.42	0.85	−0.11	0.78	−0.81	0.69	−0.71	0.36
Phenytoin								
Single dose	4.15	0.52	11.56	0.56	7.72	0.54	5.32	0.45
Steady state	2.07	1.53	7.06	1.41	3.86	1.11	1.21	0.56

**Table 4:** Population pharmacokinetics of amikacin, vancomycin, theophylline, and phenytoin in Abbottbase^®^ PKS system.

Pharmacokinetic Parameters						
Drug (Model)	Amikacin (1 CMT IV)			Vancomycin (2 CMT IV)		
Parameters	Mean (CV)	Lower	Upper	Mean (CV)	Lower	Upper
CL_slope_	0.815 (0.4)	0.3	1.7	0.75 (0.33)	0.3	1.7
CL_nr_ (mL/min/kg)	0.0417 (0.25)	0.0001	0.17	0.05 (0.2)	0.01	0.2
V_nr_ (L/kg)	0.27 (0.3)	0.15	0.65	0.21 (0.2)	0.08	0.4
k_12_ (1/h)	-	-	-	1.12 (0.25)	0.6	1.6
k_21_ (1/h)	-	-	-	0.48 (0.25)	0.2	1.0
**Drug (Model)**	**Theophylline (1 CMT oral)**			**Phenytoin (1 CMT oral)**		
**Parameters**	**Mean (CV)**	**Lower**	**Upper**	**Mean (CV)**	**Lower**	**Upper**
CL_slope_	-	-	-	0.01	-	-
CL_nr_ (mL/h/kg)	40.0 (0.5)	15.0	90.0	-	-	-
V_nr_ (L/kg)	0.5 (0.2)	0.35	0.65	0.8 (0.2)	0.3	1.4
k_a_	0.27	-	-	-	-	-
F	1	-	-	0.92	-	-
V_max_ (mg/kg/d)	-	-	-	500 (0.3)	250.0	2000.0
k_m_ (mcg/mL)	-	-	-	5.0 (0.5)	2.0	9.0
**Parameter Equations**						
**Model**	**Linear Pharmacokinetics**			**Nonlinear Pharmacokinetics**		
CL (L/h)	$CL=CL_{slope}\cdot CrCL+CL_{nr}\cdot LBW$			$CL=\frac{V_{max}}{k_{m}+C}+CL_{slope}\cdot CrCL$		
V (L)	$V=V_{nr}\cdot LBW$			$V=V_{nr}\cdot {\left(TBW/70\right)}^{0.6}$		
**Variability Equations**						
Parameters	$Parameter=Mean\cdot e^{\eta^{1}}$ $\eta^{1}∼N\left(0,\omega^{2}\right)$ $,\;\omega^{2}=\ln(CV^{2}+1)$					
Concentration	$C_{Obs}=C_{Pred}+\varepsilon_{1}$ $\varepsilon_{1}∼N\left(0,\sigma^{2}\right)$ $,\;\sigma =CV_{assay}\cdot C_{Pred}+S_{assay}$					
	$CV_{assay}=0.15$ $S_{assay}=0.25$(mg/L)			$CV_{assay}=0.1$ $S_{assay}=1.0$(mg/L)		

Abbreviations: CMT, compartment; IV, intravenous; CV, coefficient of variance; CL_slope_, rate of change in drug clearance with respect to creatinine clearance; CL_nr_, clearance independent of renal function; V_nr_, distribution volume independent of renal function; k_12_, first-order transfer rate constant from the central compartment to peripheral compartment; k_21_, first-order transfer rate constant from the peripheral compartment to central compartment; k_a_, first-order absorption rate constant; F, bioavailability; V_max_, maximum velocity; k_m_, Michaelis constant; CL, clearance; V, volume of distribution; CrCL, creatinine clearance in L/h; LBW, lean body weight in kg; TBW, total body weight in kg; C_Obs_, observed concentration; C_Pred_, predicted concentration; CV_assay_, assay coefficient of variation; S_assay_, assay sensitivity.

**Table 5:** Simulation scenario of dosage regimen and blood sampling time.

Drug		Amikacin	Vancomycin	Theophylline	Phenytoin
Dose (mg) [25,26,27,28]		500	1000	200	100
Infusion rate (mg/h)		1000	500	-	50 *
Dosing Interval (h)		8	12	12	8
Sampling time (h) [29,30,31]					
Set 1	Peak	1	2	4	2
Set 2	Trough	8	12	12	8
Set 3	Peak and trough	1, 8	2, 12	4, 12	2, 8
Set 4	Every 1 h	1 to 8	1 to 12	1 to 12	1 to 8

Notes: * zero-order absorption rate (mg/h) of phenytoin.

**Table 6:** Population pharmacokinetics of amikacin, vancomycin, theophylline, and phenytoin for external validation.

Component	Equation
**Amikacin** [32]	
Pharmacokinetic Parameters	$CL\;\left(L/h\right)=\left(1.40+1.42\cdot \left(CrCL/71.2\right)\right)\cdot e^{\eta^{1}}$ $V\;\left(L\right)=10.8+7.24\cdot \left(TBW/57\right)$
Interindividual Variability	$\eta^{1}∼N\left(0,0.303^{2}\right)$
Residual errors	$C_{Obs}=C_{\mathrm{Pr}ed}\cdot \left(1+\varepsilon_{1}\right)$ $\varepsilon_{1}∼N\left(0,0.307^{2}\right)$
**Vancomycin** [33]	
Pharmacokinetic Parameters	$CL\;\left(L/h\right)=\left(2.82\cdot {\left(CrCL/72\right)}^{0.836}\right)\cdot e^{\eta^{1}}$ $V_{c}\;\left(L\right)=31.8$ $Q\;\left(L/h\right)=11.7$ $V_{p}\;\left(L\right)=\left(75.4\cdot \left(TBW/60\right)\right)\cdot e^{\eta^{2}}$
Interindividual Variability	$\eta^{1}∼N\left(0,0.828^{2}\right)$ $\mathrm{and}\;\eta^{2}∼N\left(0,0.466^{2}\right)$
Residual errors	$C_{Obs}=C_{\mathrm{Pr}ed}\cdot \left(1+\varepsilon_{1}\right)$ $\varepsilon_{1}∼N\left(0,0.253^{2}\right)$
**Theophylline** [34]	
Pharmacokinetic Parameters	$k_{a}\;\left(1/h\right)=0.0773$ $CL/F\;\left(L/h/kg\right)=\left(0.0539\cdot 0.876^{eldery}\right)\cdot e^{\eta^{1}}$* $V/F\;\left(L/kg\right)=0.320\cdot e^{\eta^{2}}$
Interindividual Variability	$\eta^{1}∼N\left(0,0.313^{2}\right)$ $\mathrm{and}\;\eta^{2}∼N\left(0,0.284^{2}\right)$
Residual errors	$C_{Obs}=C_{\mathrm{Pr}ed}\cdot e^{\varepsilon_{1}}$ $\varepsilon_{1}∼N\left(0,0.178^{2}\right)$
**Phenytoin** [35]	
Pharmacokinetic Parameters	$V_{max}\;\left(mg/kg/d\right)=(9.80\cdot 42\cdot \left(TBW/42{)}^{0.463}\right)\cdot \left(1+\eta^{1}\right)$ $k_{m}\;\left(mcg/mL\right)=9.19\cdot \left(1+\eta^{2}\right)$ $V\;\left(L/kg\right)=1.23\cdot \left(1+\eta^{3}\right)$
Interindividual Variability	$\eta^{1}∼N\left(0,0.150^{2}\right)$ $,\;\eta^{2}∼N\left(0,0.306^{2}\right)$ $,\;\mathrm{and}\;\eta^{3}∼N\left(0,0.433^{2}\right)$
Residual errors	$C_{Obs}=C_{\mathrm{Pr}ed}\cdot \left(1+\varepsilon_{1}\right)$ $\varepsilon_{1}∼N\left(0,0.181^{2}\right)$

Notes: * elderly is a dichotomous covariate coded as elderly = 0 if age <65, and elderly = 1 if age ≥65. Abbreviations: CL, clearance; V, volume of distribution; C_Obs_, observed concentration; C_Pred_, predicted concentration; V_c_, central volume of distribution; Q, intercompartmental clearance; V_p_, peripheral volume of distribution; k_a_, first-order absorption rate constant; V_max_, maximum velocity; k_m_, Michaelis constant; CrCL, creatinine clearance in mL/min; TBW, total body weight in kg.

## Data Availability

HMCtdm is provided in a repository at https://github.com/SikSo1897/hmctdm/tree/develop (accessed on 1 January 2022).

## Supplementary Materials

The following are available online at https://www.mdpi.com/article/10.3390/ph15020127/s1, Supplementary File S1: The results for internal validation of PK parameters; Supplementary File S2: The results for external validation of PK parameters; Supplementary File S3: The results for internal validation of PK parameters using MAP estimation.
