# Supplementary material for: Development and Validation of Open-Source R Package HMCtdm for Therapeutic Drug Monitoring
Source: Pharmaceuticals (Basel). 2022 Jan 21;15(2):127. doi: 10.3390/ph15020127 (PMC8875672; doi:10.3390/ph15020127)
Supplement: Supplementary file 1 [file pharmaceuticals-15-00127-s001.zip › [Supplementary File S1]_The results for Internal validation_English_edited.pdf]

Table S1. Internal validation data estimation of amikacin pharmacokinetic parameters

Sampling time	Peak		Trough		Peak and trough		Every 1 h	
	MPE	RMSE	MPE	RMSE	MPE	RMSE	MPE	RMSE
**Single dose**								
CL_{slope}	8.32	0.30	2.97	0.14	2.94	0.12	1.37	0.06
CL_{nr} (mL/min/kg)	3.00	0.01	3.02	0.01	3.02	0.01	2.90	0.01
V_{nr} (L/kg)	1.70	0.04	2.77	0.08	1.99	0.04	1.14	0.03
**Steady-state**								
CL_{slope}	3.80	0.21	1.37	0.13	1.44	0.09	0.95	0.05
CL_{nr} (mL/min/kg)	3.06	0.01	3.07	0.01	3.09	0.01	3.05	0.01
V_{nr} (L/kg)	1.00	0.08	1.98	0.08	1.59	0.06	0.91	0.04

Abbreviations: MPE, mean percent error; RMSE, root mean squared error; CL_{slope}, rate of change in drug clearance with respect to creatinine clearance; CL_{nr}, clearance independent of renal function; V_{nr}, distribution volume independent of renal function.

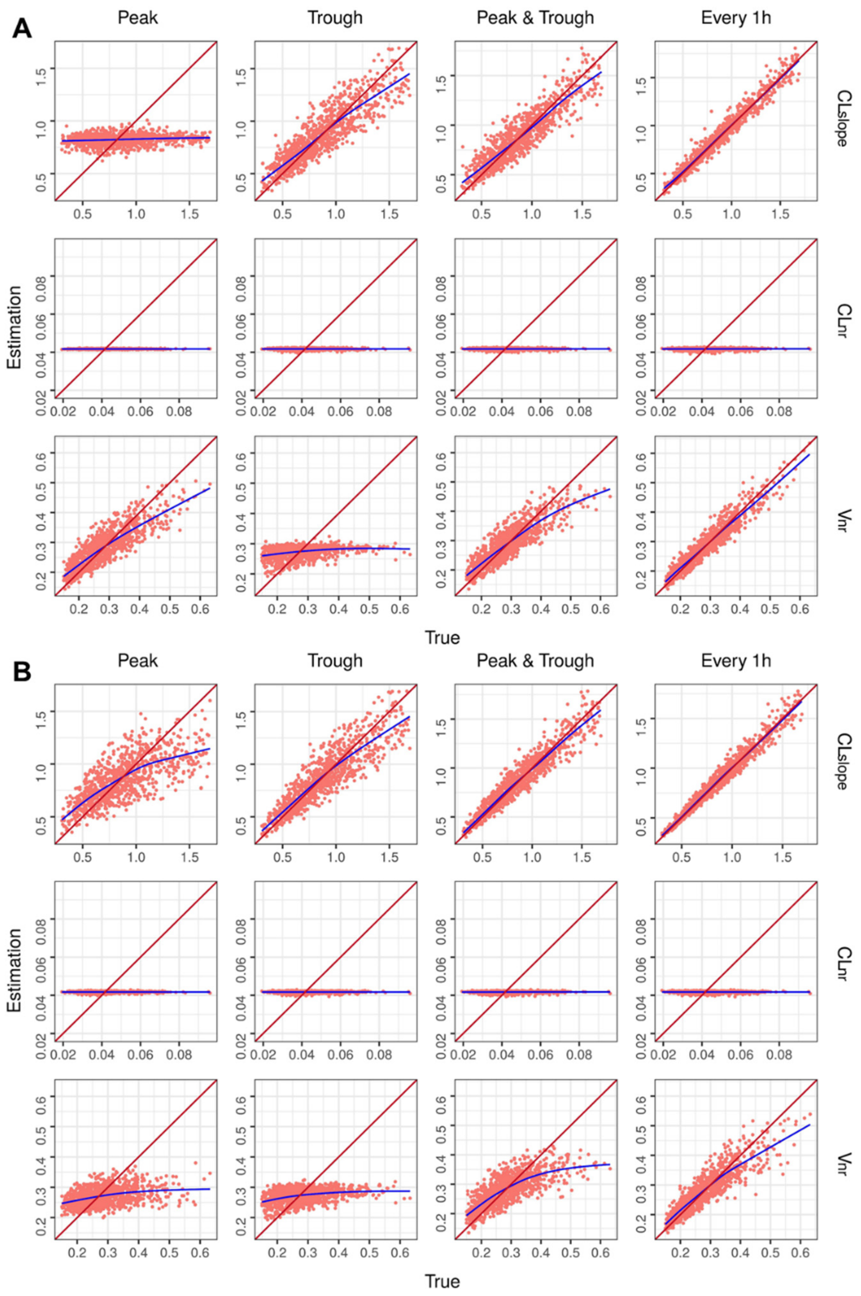

Figure S1. Graphs representing the estimated amikacin parameters versus true values under each internal validation scenario. The identity line is shown in red, and a trend line in blue has been drawn for each model: (**A**) single dose and (**B**) steady state. **Abbreviations**: CL_{slope}, rate of change in drug clearance with respect to creatinine clearance; CL_{nr}, clearance independent of renal function; V_{nr}, distribution volume independent of renal function.

Table S2. Internal validation data estimation of vancomycin pharmacokinetic parameters

Sampling time	Peak		Trough		Peak and trough		Every 1 h	
	MPE	RMSE	MPE	RMSE	MPE	RMSE	MPE	RMSE
**Single dose**								
CL_{slope}	4.21	0.25	3.34	0.16	3.57	0.16	2.96	0.12
CL_{nr} (mL/min/kg)	1.07	0.01	1.09	0.01	1.10	0.01	1.11	0.01
V_{nr} (L/kg)	0.69	0.04	1.93	0.04	0.51	0.04	0.14	0.03
k_{12} (1/h)	4.28	0.19	6.08	0.22	4.01	0.19	4.42	0.19
k_{21} (1/h)	3.37	0.11	2.28	0.12	2.93	0.11	1.45	0.09
**Steady-state**								
CL_{slope}	0.47	0.15	1.78	0.11	0.53	0.08	0.68	0.04
CL_{nr} (mL/min/kg)	1.11	0.01	1.16	0.01	1.10	0.01	1.21	0.01
V_{nr} (L/kg)	1.58	0.04	1.51	0.04	1.08	0.04	0.42	0.04
k_{12} (1/h)	5.43	0.22	5.58	0.22	4.56	0.20	4.53	0.20
k_{21} (1/h)	3.05	0.12	2.91	0.12	3.17	0.12	2.64	0.10

Abbreviations: MPE, mean percent error; RMSE, root mean squared error; CL_{slope}, rate of change in drug clearance with respect to creatinine clearance; CL_{nr}, clearance independent of renal function; V_{nr}, distribution volume independent of renal function; k_{12}, first-order transfer rate constant from the central compartment to peripheral compartment; k_{21}, first-order transfer rate constant from the peripheral compartment to central compartment.

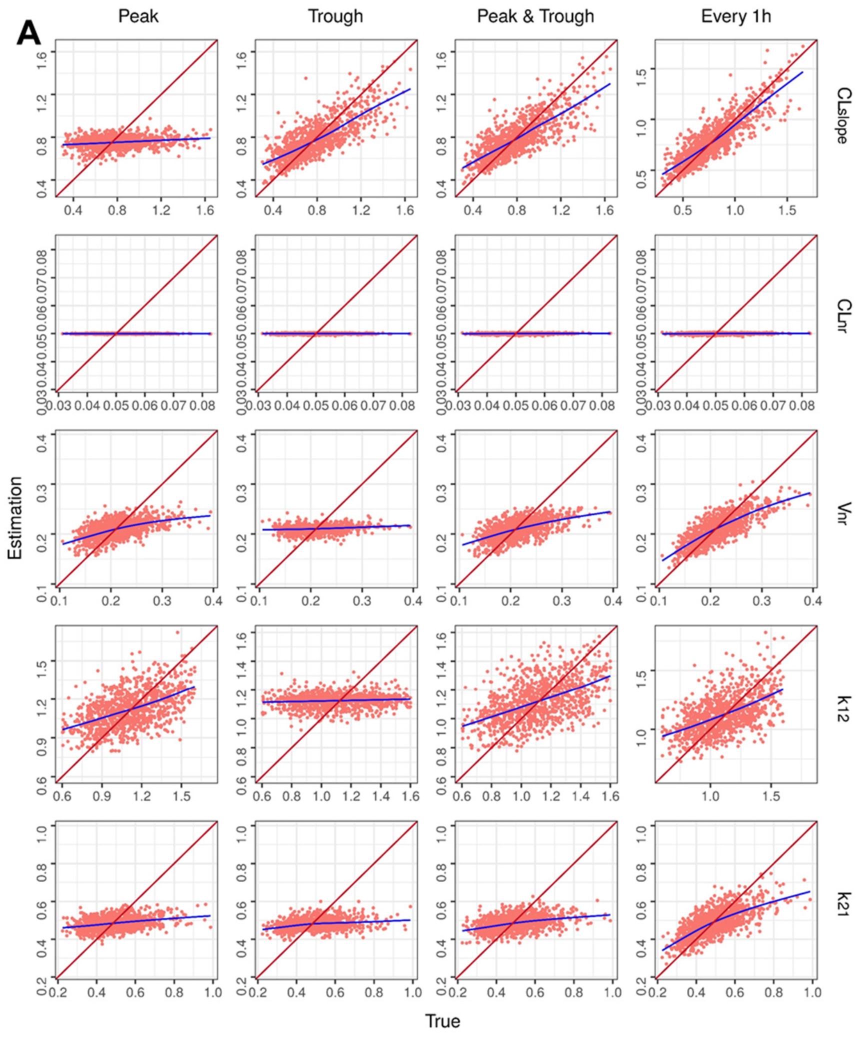

Figure S2. *Cont*.

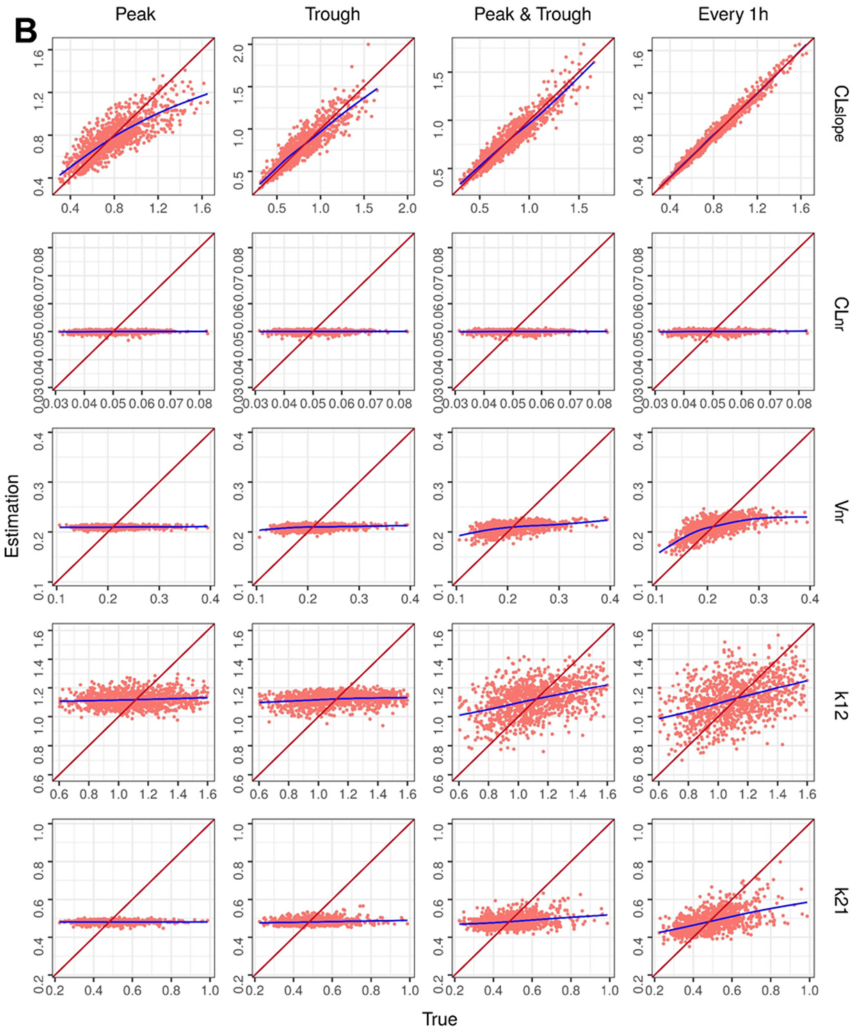

Figure S2. Graphs representing the estimated vancomycin parameters versus true values under each internal validation scenario. The identity line is shown in red, and a trend line in blue is drawn for each model: (**A**) single dose and (**B**) steady state.

Abbreviations: CL_{slope}, rate of change in drug clearance with respect to creatinine clearance; CL_{nr}, clearance independent of renal function; V_{nr}, distribution volume independent of renal function; k_{12}, first-order transfer rate constant from the central compartment to peripheral compartment; k_{21}, first-order transfer rate constant from the peripheral compartment to central compartment.

Table S3. Internal validation data estimation of theophylline pharmacokinetic parameters

Sampling time	Peak		Trough		Peak and trough		Every 1 h	
	MPE	RMSE	MPE	RMSE	MPE	RMSE	MPE	RMSE
**Single dose**								
CL_{nr} (mL/h/kg)	1.53	5.82	1.43	5.23	1.25	5.30	1.22	4.62
V_{nr} (L/kg)	2.31	0.06	2.72	0.07	2.08	0.06	1.69	0.04
**Steady-state**								
CL_{nr} (mL/h/kg)	1.08	4.55	1.11	4.20	1.20	3.55	0.71	1.80
V_{nr} (L/kg)	2.45	0.07	2.47	0.07	2.33	0.07	2.15	0.07

Abbreviations: MPE, mean percent error; RMSE, root mean squared error; CL_{nr}, clearance independent of renal function; V_{nr}, distribution volume independent of renal function.

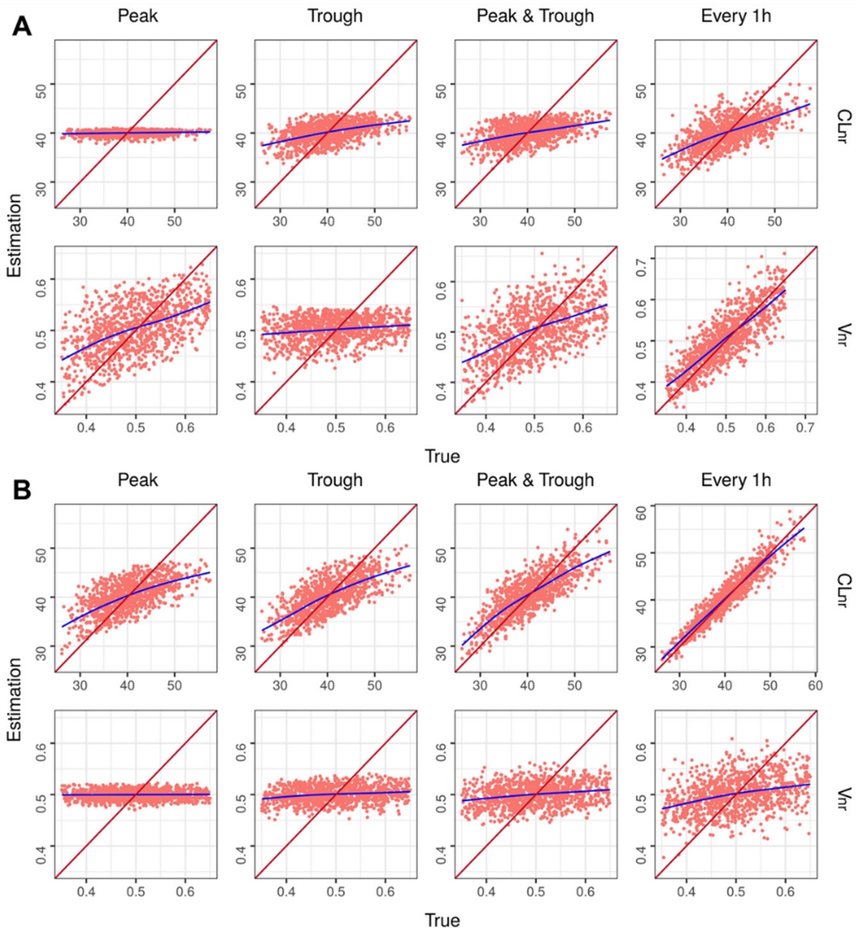

Figure S3. Graphs representing the estimated theophylline parameters versus true values under each internal validation scenario. The identity line is shown in red, and a trend line in blue is drawn for each model: (**A**) single dose and (**B**) steady state. **Abbreviations:** CL_{nr}, clearance independent of renal function; V_{nr}, distribution volume independent of renal function.

Table S4. Internal validation data estimation of phenytoin pharmacokinetic parameters

Sampling time	Peak		Trough		Peak and trough		Every 1 h	
	MPE	RMSE	MPE	RMSE	MPE	RMSE	MPE	RMSE
**Single dose**								
V_{max} (mg/kg/d)	3.31	135.33	3.34	134.71	3.42	134.69	3.55	132.14
k_m (mcg/mL)	10.25	2.04	10.10	2.03	10.07	2.04	9.73	2.02
V_{nr} (L/kg)	1.39	0.14	1.74	0.14	1.67	0.14	2.06	0.13
**Steady-state**								
V_{max} (mg/kg/d)	0.35	95.75	0.05	95.61	0.29	92.65	0.95	86.49
k_m (mcg/mL)	9.17	1.89	9.18	1.88	8.63	1.85	7.62	1.82
V_{nr} (L/kg)	1.20	0.15	1.06	0.14	0.91	0.14	0.68	0.14

Abbreviations: MPE, mean percent error; RMSE, root mean squared error; V_{max}, maximum velocity; k_m, Michaelis constant; V_{nr}, distribution volume independent of renal function.

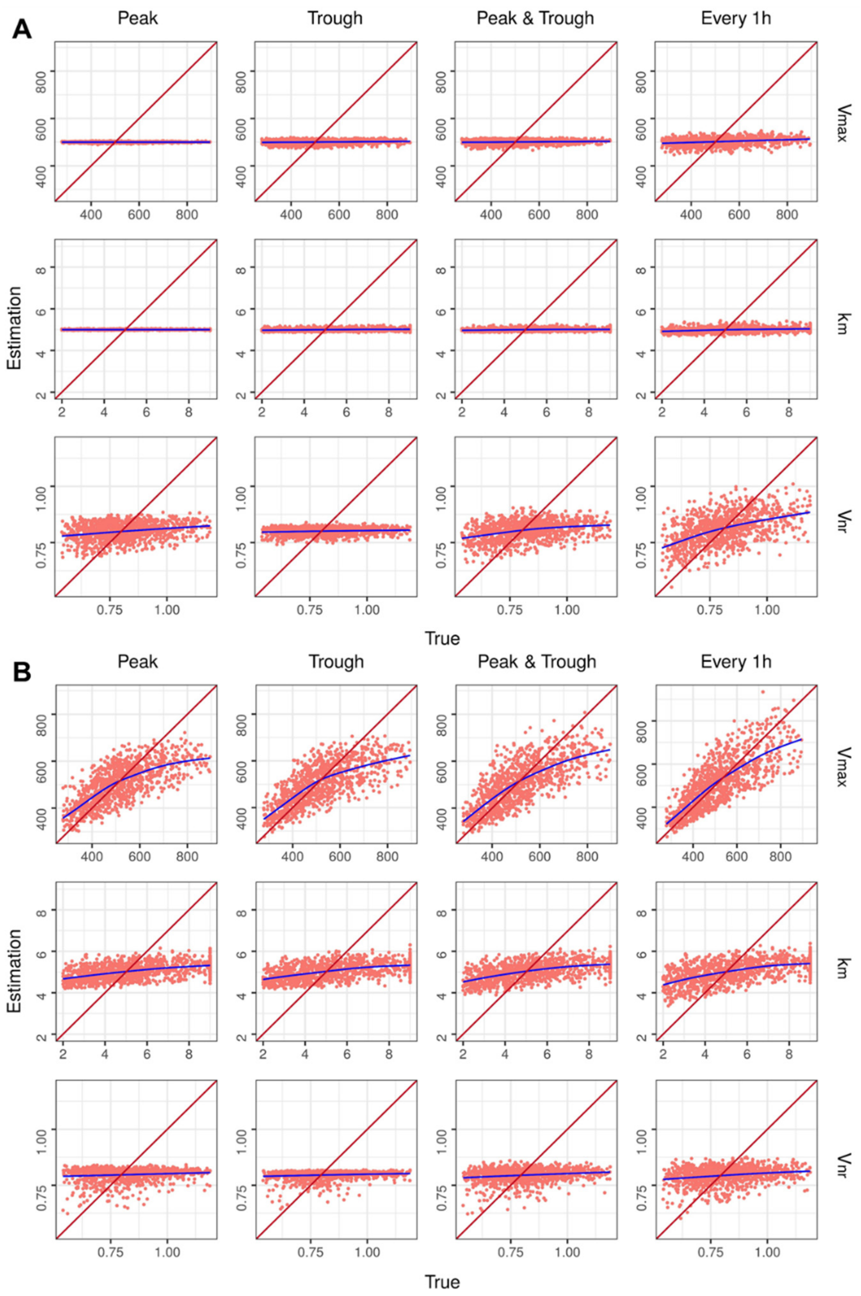

Figure S4. Graphs representing the estimated phenytoin parameters versus true values under each internal validation scenario. The identity line is shown in red, and a trend line in blue is drawn for each model: (**A**) single dose and (**B**) steady state. **Abbreviations:** V_{max}, maximum velocity; k_m, Michaelis constant; V_{nr}, distribution volume independent of renal function.
