# Supplementary material for: Development and Validation of Open-Source R Package HMCtdm for Therapeutic Drug Monitoring
Source: Pharmaceuticals (Basel). 2022 Jan 21;15(2):127. doi: 10.3390/ph15020127 (PMC8875672; doi:10.3390/ph15020127)
Supplement: Supplementary file 1 [file pharmaceuticals-15-00127-s001.zip › [Supplementary File S2]_The results for External validation_English_edited.pdf]

Table S5. External validation data estimation of amikacin pharmacokinetic parameters

Sampling time	Peak		Trough		Peak and trough		Every 1 h	
	**MPE**	**RMSE**	**MPE**	**RMSE**	**MPE**	**RMSE**	**MPE**	**RMSE**
**Single dose**								
CL (L/h)	17.93	0.93	-2.11	0.34	1.83	0.34	1.34	0.22
V (L)	-5.24	2.49	-14.64	3.15	-1.60	2.48	-1.51	1.75
**Steady-state**								
CL (L/h)	17.00	0.70	-4.71	0.29	-1.24	0.24	-0.49	0.14
V (L)	-18.35	3.91	-14.43	3.31	-6.74	2.83	-4.83	2.53

Abbreviations: MPE, mean percent error; RMSE, root mean squared error; CL, clearance; V, volume of distribution.

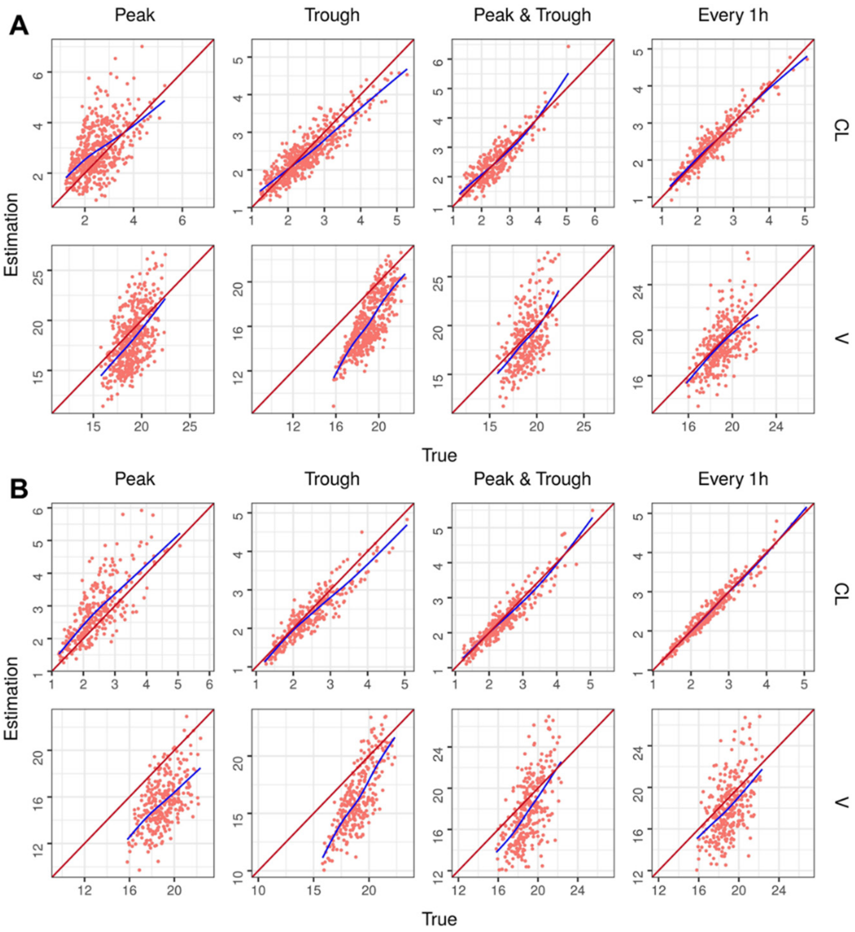

Figure S5. Graphs representing the estimated amikacin parameters versus true values in each external validation scenario. The identity line is shown in red, and a trend line in blue is drawn for each model: (**A**) single dose and (**B**) steady state. **Abbreviations:** CL, clearance; V, volume of distribution.

Table S6. External validation data estimation of vancomycin pharmacokinetic parameters

Sampling time	Peak		Trough		Peak and trough		Every 1 h	
	MPE	RMSE	MPE	RMSE	MPE	RMSE	MPE	RMSE
**Single dose**								
CL (L/h)	72.84	2.12	115.45	1.93	121.10	1.99	173.72	2.81
V_c (L)	–60.05	19.16	–62.17	19.84	–60.93	19.44	–56.13	17.92
k_{12} (1/h)	263.78	0.99	210.68	0.78	244.90	0.92	231.08	0.88
k_{21} (1/h)	228.77	0.29	243.64	0.30	227.73	0.29	211.71	0.27
**Steady-state**								
CL (L/h)	11.33	1.19	-3.43	1.14	–3.72	0.90	–4.19	0.41
V_c (L)	–62.55	19.96	–61.98	19.79	–61.57	19.65	–58.82	18.78
k_{12} (1/h)	202.43	0.75	209.86	0.77	221.82	0.83	241.30	0.90
k_{21} (1/h)	257.83	0.32	255.62	0.32	246.66	0.31	242.22	0.31

Abbreviations: MPE, mean percent error; RMSE, root mean squared error; CL, clearance; V_c, central volume of distribution; k_{12}, first-order transfer rate constant from the central compartment to peripheral compartment; k_{21}, first-order transfer rate constant from the peripheral compartment to central compartment.

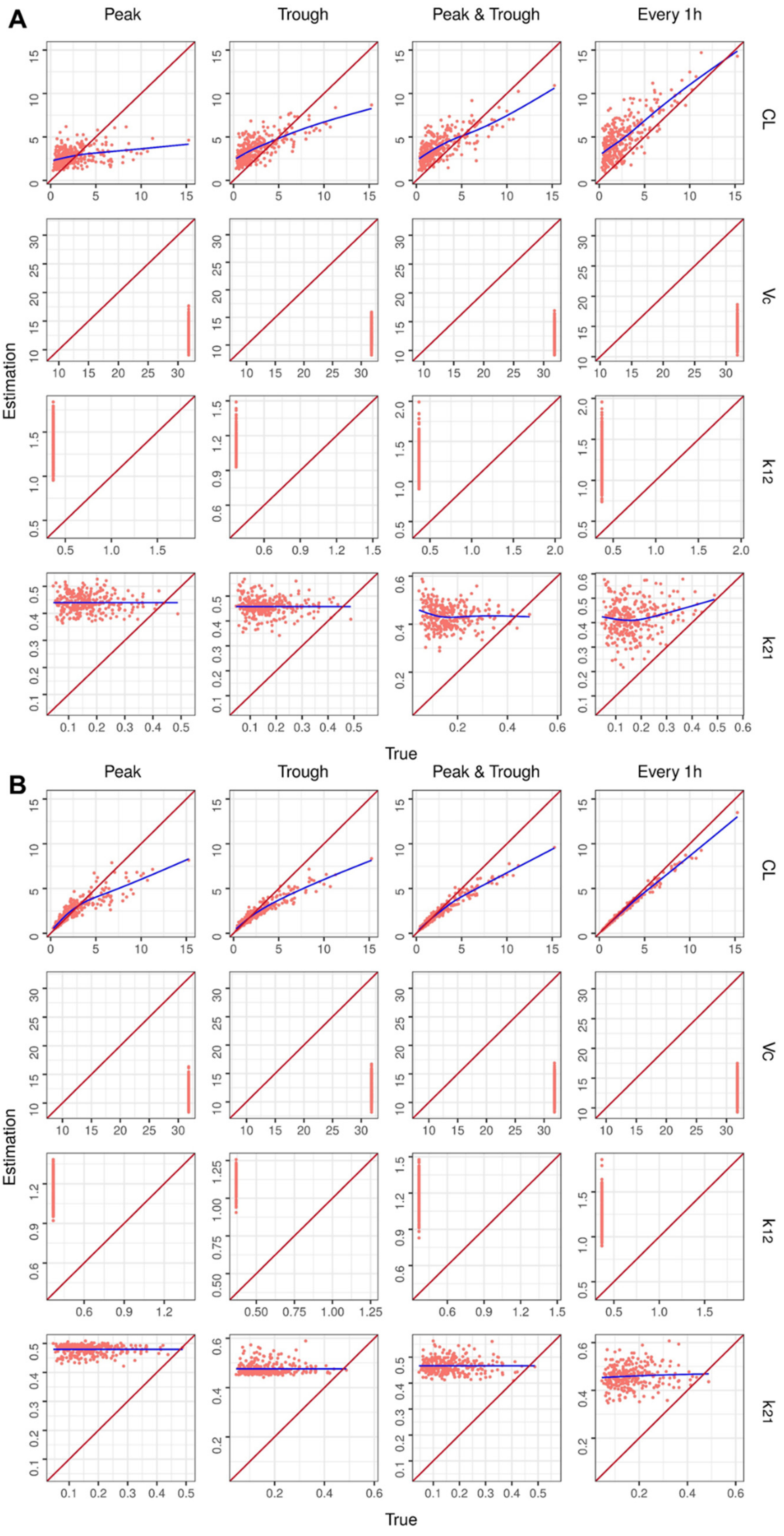

Figure S6. Graphs representing the estimated vancomycin parameters versus true values under each external validation scenario. The identity line is shown in red, and a trend line in blue is drawn for each model: (**A**) single dose and (**B**) steady state. **Abbreviations:** CL, clearance; V_c, central volume of distribution; k_{12}, first-order transfer rate constant from the central compartment to peripheral compartment; k_{21}, first-order transfer rate constant from the peripheral compartment to central compartment.

Table S7. External validation data estimation of theophylline pharmacokinetic parameters

Sampling time	Peak		Trough		Peak and trough		Every 1 h	
	MPE	RMSE	MPE	RMSE	MPE	RMSE	MPE	RMSE
**Single dose**								
CL/F (L/h)	–25.03	1.36	–22.04	1.25	–22.00	5.30	1.22	4.62
V/F (L)	75.12	14.67	52.38	10.58	84.64	0.06	1.69	0.04
**Steady-state**								
CL/F (L/h)	–19.41	1.13	–22.62	1.14	–17.68	3.55	0.71	1.80
V/F (L)	47.39	9.75	40.13	8.67	45.31	0.07	2.15	0.07

Abbreviations: MPE, mean percent error; RMSE, root mean squared error; CL/F, apparent clearance; V/F, apparent volume of distribution.

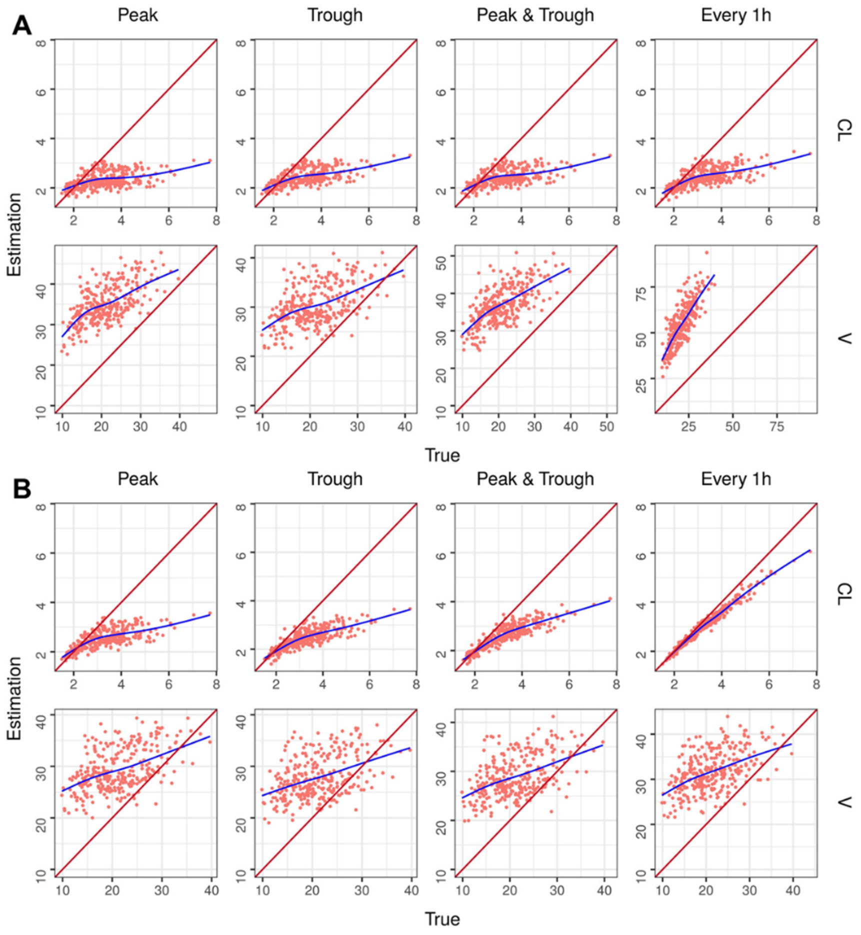

Figure S7. Graphs representing the estimated theophylline parameters versus true values under each external validation scenario. The identity line is shown in red, and a trend line in blue is drawn for each model: (**A**) single dose and (**B**) steady state. **Abbreviations:** CL, apparent clearance; V, apparent volume of distribution.

Table S8. External validation data estimation of phenytoin pharmacokinetic parameters

Sampling time	Peak		Trough		Peak and trough		Every 1 h	
	MPE	RMSE	MPE	RMSE	MPE	RMSE	MPE	RMSE
**Single dose**								
V_{max} (mg/kg/d)	4.47	69.60	5.04	69.41	5.42	70.88	8.39	79.02
k_m (mcg/mL)	–41.83	4.77	–42.03	4.77	–42.18	4.78	–43.27	4.86
V_{nr} (L/kg)	–28.13	39.01	–29.75	41.90	–27.39	38.23	–20.91	28.65
**Steady-state**								
V_{max} (mg/kg/d)	–4.16	71.86	–6.43	78.40	–6.58	79.82	–7.82	83.88
k_m (mcg/mL)	–39.74	4.52	–39.03	4.47	–38.98	4.45	–38.68	4.41
V_{nr} (L/kg)	–31.35	43.04	–30.38	42.75	–30.52	42.44	–30.81	42.33

Abbreviations: MPE, mean percent error; RMSE, root mean squared error; V_{max}, maximum velocity; k_m, Michaelis constant; V_{nr}, distribution volume independent of renal function.

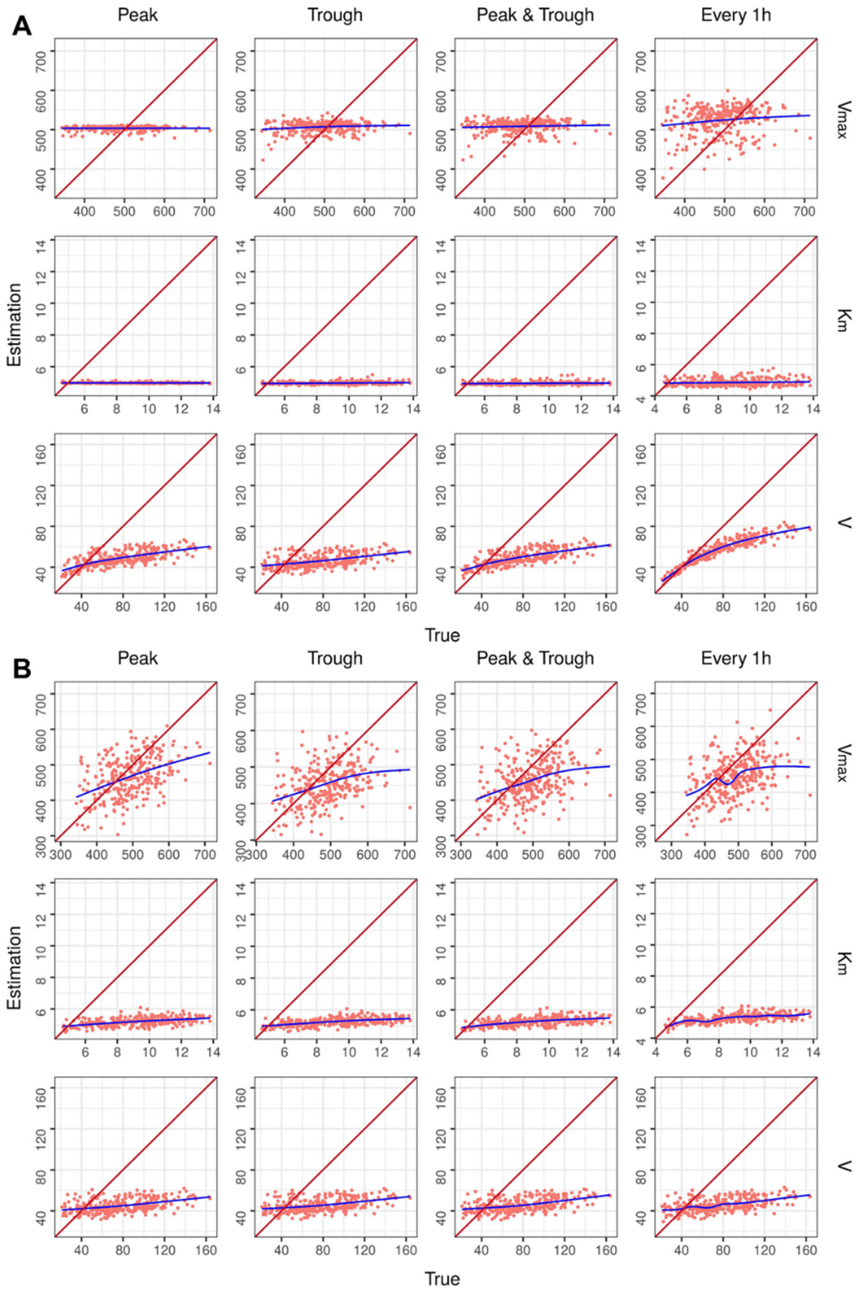

Figure S8. Graphs representing the estimated phenytoin parameters versus true values under each external validation scenario. The identity line is shown in red, and a trend line in blue has been drawn for each model: (**A**) single dose and (**B**) steady state. **Abbreviations:** V_{max}, maximum velocity; k_m, Michaelis constant; V_{nr}, distribution volume independent of renal function.
