# Supplementary material for: Development and Validation of Open-Source R Package HMCtdm for Therapeutic Drug Monitoring
Source: Pharmaceuticals (Basel). 2022 Jan 21;15(2):127. doi: 10.3390/ph15020127 (PMC8875672; doi:10.3390/ph15020127)
Supplement: Supplementary file 1 [file pharmaceuticals-15-00127-s001.zip › [Supplementary File S3]_The results for Internal validation with MAP estimation_English_edited.pdf]

Table S9. Internal validation data estimation of amikacin pharmacokinetic parameters using MAP estimation

Sampling time	Peak		Trough		Peak and trough		Every 1 h	
	MPE	RMSE	MPE	RMSE	MPE	RMSE	MPE	RMSE
**Single dose**								
CL_{slope}	7.61	0.30	7.75	0.15	7.27	0.13	2.58	0.06
CL_{nr} (mL/min/kg)	3.01	0.01	2.95	0.01	2.91	0.01	2.75	0.01
V_{nr} (L/kg)	4.18	0.04	–2.67	0.08	1.52	0.04	0.79	0.03
**Steady-state**								
CL_{slope}	1.00	0.21	2.99	0.13	3.61	0.09	1.35	0.06
CL_{nr} (mL/min/kg)	2.85	0.01	2.78	0.01	2.78	0.01	2.70	0.01
V_{nr} (L/kg)	2.97	0.08	–0.69	0.08	0.06	0.06	0.30	0.04

Abbreviations: MPE, mean percent error; RMSE, root mean squared error; CL_{slope}, rate of change in drug clearance with respect to creatinine clearance; CL_{nr}, clearance independent of renal function; V_{nr}, distribution volume independent of renal function.

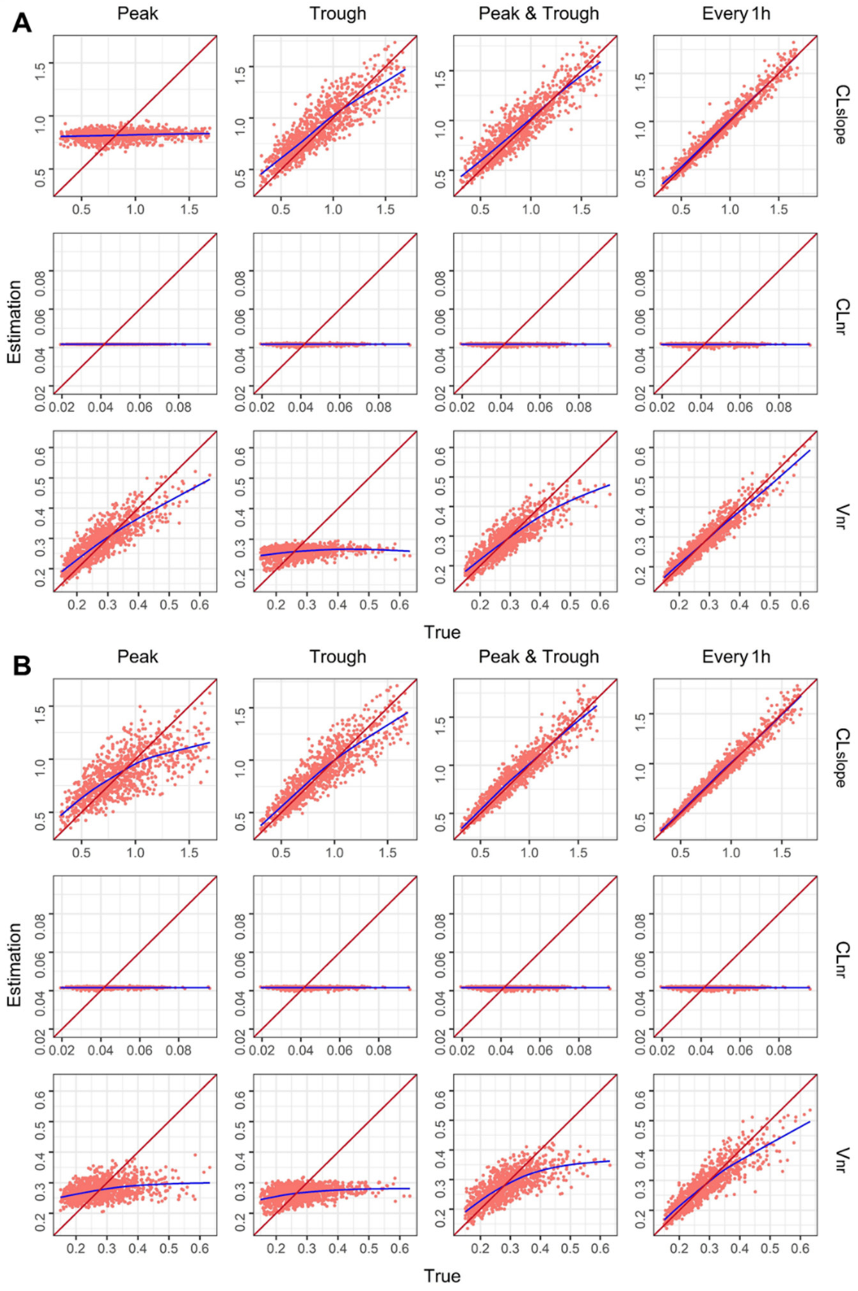

Figure S9. Graphs representing the estimated amikacin parameters versus true values under each internal validation scenario using MAP estimation. The identity line is shown in red, and a trend line in blue is drawn for each model: (**A**) single dose and (**B**) steady state. **Abbreviations**: CL_{slope}, rate of change in drug clearance with respect to creatinine clearance; CL_{nr}, clearance independent of renal function; V_{nr}, distribution volume independent of renal function.

Table S10. Internal validation data estimation of vancomycin pharmacokinetic parameters using MAP estimation

Sampling time	Peak		Trough		Peak and trough		Every 1 h	
	MPE	RMSE	MPE	RMSE	MPE	RMSE	MPE	RMSE
**Single dose**								
CL_{slope}	4.12	0.25	8.61	0.17	7.67	0.16	5.33	0.13
CL_{nr} (mL/min/kg)	1.08	0.01	1.12	0.01	1.11	0.01	1.06	0.01
V_{nr} (L/kg)	1.42	0.04	1.08	0.04	–0.10	0.04	–0.15	0.03
k_{12} (1/h)	6.96	0.20	5.38	0.22	5.12	0.19	4.73	0.19
k_{21} (1/h)	1.69	0.12	2.48	0.12	2.63	0.11	2.11	0.09
**Steady-state**								
CL_{slope}	1.85	0.15	3.67	0.11	2.49	0.08	1.21	0.04
CL_{nr} (mL/min/kg)	1.00	0.01	1.02	0.01	0.99	0.01	0.96	0.01
V_{nr} (L/kg)	1.70	0.04	0.46	0.04	–0.38	0.04	–0.41	0.03
k_{12} (1/h)	6.41	0.22	4.55	0.22	4.07	0.20	4.26	0.20
k_{21} (1/h)	2.52	0.12	3.88	0.12	3.83	0.12	3.32	0.10

Abbreviations: CL_{slope}, rate of change in drug clearance with respect to creatinine clearance; CL_{nr}, clearance independent of renal function; V_{nr}, distribution volume independent of renal function; k_{12}, first-order transfer rate constant from the central compartment to peripheral compartment; k_{21}, first-order transfer rate constant from the peripheral compartment to central compartment.

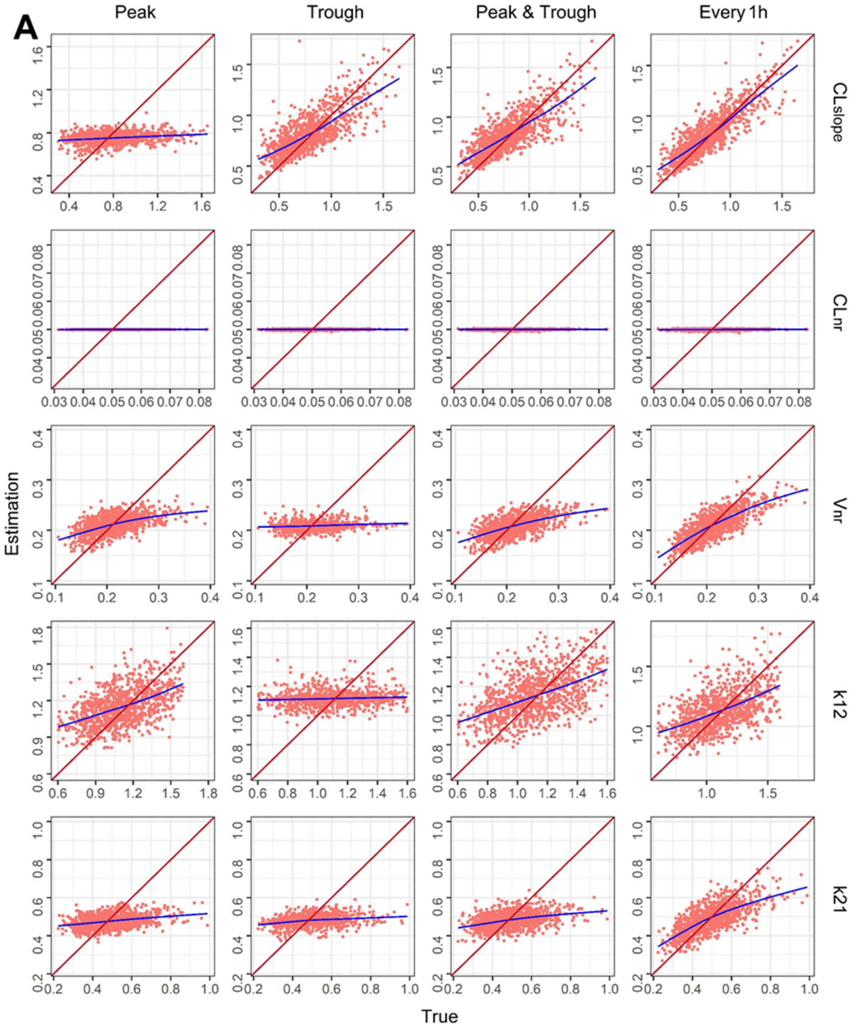

Figure S2. *Cont*.

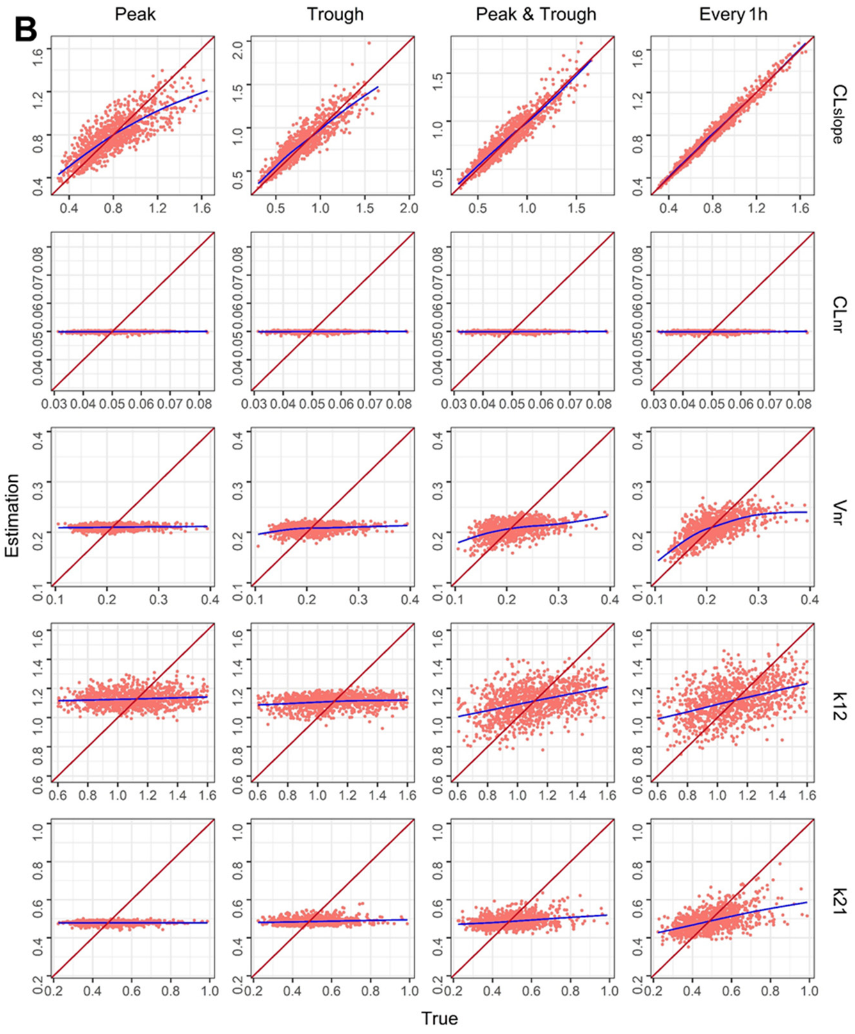

Figure S10. Graphs representing the estimated vancomycin parameters versus true values under each internal validation scenario using MAP estimation. The identity line is shown in red, and a trend line in blue is drawn for each model: (**A**) single dose and (**B**) steady state. **Abbreviations:** CL_{slope}, rate of change in drug clearance with respect to creatinine clearance; CL_{nr}, clearance independent of renal function; V_{nr}, distribution volume independent of renal function; k_{12}, first-order transfer rate constant from the central compartment to peripheral compartment; k_{21}, first-order transfer rate constant from the peripheral compartment to central compartment.

Table S11. Internal validation data estimation of theophylline pharmacokinetic parameters using MAP estimation

Sampling time	Peak		Trough		Peak and trough		Every 1 h	
	MPE	RMSE	MPE	RMSE	MPE	RMSE	MPE	RMSE
**Single dose**								
CL_{nr} (mL/h/kg)	1.44	5.83	1.78	5.24	1.47	5.30	1.56	4.62
V_{nr} (L/kg)	2.89	0.06	2.94	0.07	2.58	0.06	1.85	0.04
**Steady-state**								
CL_{nr} (mL/h/kg)	1.25	4.54	1.79	4.21	1.76	3.56	0.95	1.82
V_{nr} (L/kg)	2.67	0.07	1.81	0.07	1.80	0.07	1.49	0.07

Abbreviations: MPE, mean percent error; RMSE, root mean squared error; CL_{nr}, clearance independent of renal function; V_{nr}, distribution volume independent of renal function.

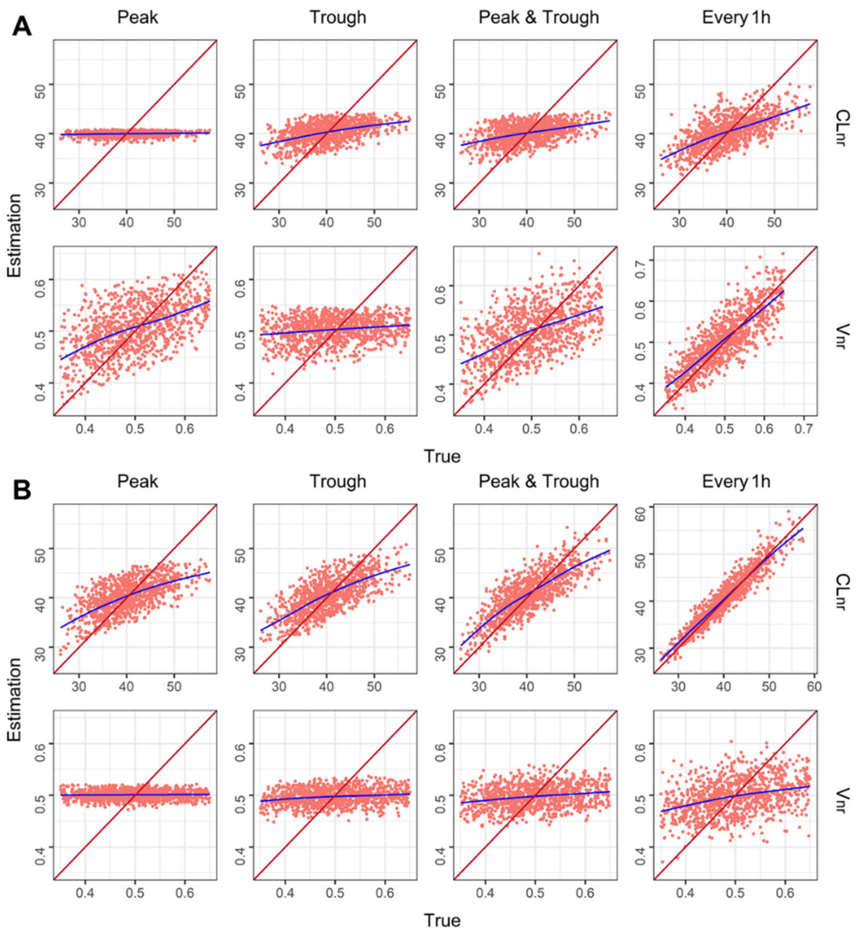

Figure S11. Graphs representing the estimated theophylline parameters versus true values under each internal validation scenario using MAP estimation. The identity line is shown in red, and a trend line in blue is drawn for each model: (**A**) single dose and (**B**) steady state. **Abbreviations:** CL_{nr}, clearance independent of renal function; V_{nr}, distribution volume independent of renal function.

Table S12. Internal validation data estimation of phenytoin pharmacokinetic parameters using MAP estimation

Sampling time	Peak		Trough		Peak and trough		Every 1 h	
	MPE	RMSE	MPE	RMSE	MPE	RMSE	MPE	RMSE
**Single dose**								
V_{max} (mg/kg/d)	3.15	135.41	3.45	134.71	3.54	135.05	3.86	130.56
k_m (mcg/mL)	10.57	2.04	9.69	2.01	9.62	2.03	8.30	1.98
V_{nr} (L/kg)	0.81	0.14	1.33	0.14	0.86	0.14	0.37	0.13
**Steady-state**								
V_{max} (mg/kg/d)	0.67	94.99	0.27	95.41	0.23	93.43	0.21	86.28
k_m (mcg/mL)	6.96	1.81	7.28	1.78	6.85	1.73	6.41	1.74
V_{nr} (L/kg)	0.98	0.14	0.54	0.15	0.47	0.14	0.30	0.14

Abbreviations: MPE, mean percent error; RMSE, root mean squared error; V_{max}, maximum velocity; k_m, Michaelis constant; V_{nr}, distribution volume independent of renal function.

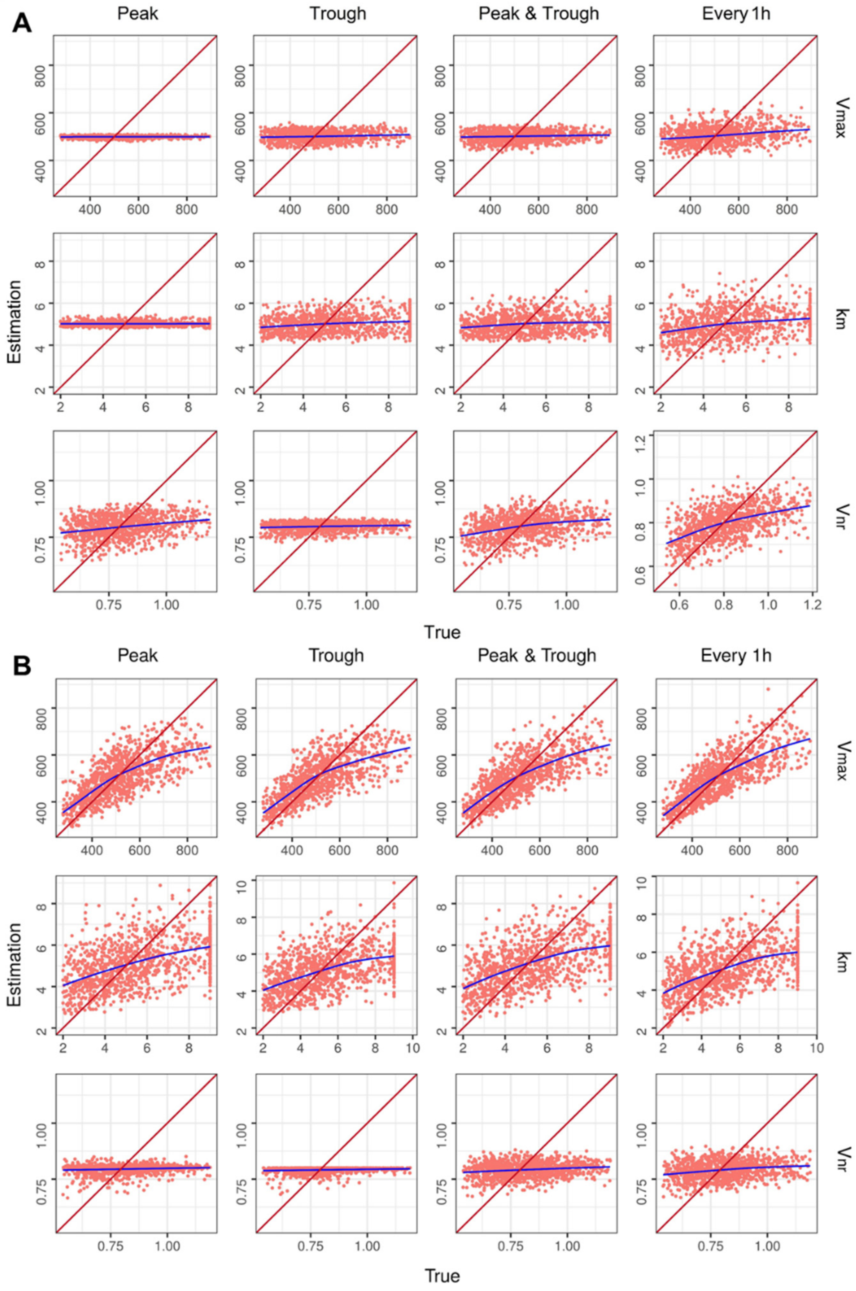

Figure S12. Graphs representing the estimated phenytoin parameters versus true values under each internal validation scenario using MAP estimation. The identity line is shown in red, and a trend line in blue is drawn for each model: (**A**) single dose and (**B**) steady state. **Abbreviations:** V_{max}, maximum velocity; k_m, Michaelis constant; V_{nr}, distribution volume independent of renal function.
